# Synthesis, antimycobacterial evaluation, and molecular docking study of 1,2,4-triazole derivatives

**DOI:** 10.1080/14756366.2023.2229070

**Published:** 2023-06-29

**Authors:** Meng-Yu Xia, Yu-Xiang Cai, Jun-Xian Chen, Xin Zhao, Hong-Mei Dong, Zai-Chang Yang

**Affiliations:** College of Pharmacy, Guizhou University, Guiyang, China

**Keywords:** *Mycobacterium tuberculosis*, KatG, inhibitor, triazole derivatives

## Abstract

Fifteen 1,2,4-triazole derivatives were synthesised in this study and their MIC values against *Mycobacterium tuberculosis* (*Mtb*) ranged from 2 to 32 μg/mL. Furthermore, their antimycobacterial activity was positively correlated with the KatG enzyme docking score. Among the 15 compounds, compound **4** showed the strongest bactericidal activity with an MIC of 2 μg/mL. The selectivity index of compound **4** is more than 10, indicating that the compound has low toxicity to animal cells and has the potential to become a drug. Molecular docking indicates that compound **4** can bind firmly to the *Mtb* KatG active site. The experimental results showed that compound **4** inhibited *Mtb* KatG and caused the accumulation of ROS in *Mtb* cells. We speculate that compound **4** causes the accumulation of ROS by inhibiting KatG, and ROS produces oxidative destruction, leading to the death of *Mtb*. This study provides a new idea for the development of novel anti-*Mtb* drugs.

## Introduction

*Mycobacterium tuberculosis* (*Mtb*) infection is known to be the cause of tuberculosis (TB), with 10.6 million infections reported in 2021, claiming about 1.6 million lives^1^. The number of multidrug-resistant and extensively drug-resistant strains of *Mtb* has increased under the pressure of anti-TB drug selection. These drug-resistant *Mtb* strains are associated with low cure rates, high mortality rates, and significantly increased treatment costs. More worryingly, fully drug-resistant strains are being isolated in clinical cases^2^. In recent years, three new drugs, bedaquiline, delamanid, and pretomanid, have been approved for the treatment of multidrug-resistant TB. However, no sooner have these drugs entered clinical use than strains of *Mtb* resistant to some of these molecules have been reported^3^^,^^4^. Therefore, there remains an urgent need to rapidly develop novel TB drugs and to continuously replenish the research pipeline.

The targets of some TB drugs have been elucidated. The bactericidal antibiotic killing mechanisms are currently attributed to the drug–target interactions. However, the details of drug–target interactions leading to bacterial death are not fully understood^5^. With the rapid spread of antibiotic-resistant strains, future antimicrobial drug development will require a better understanding of the specific sequence of events leading to cell death. Molecules that stimulate the formation of hydroxyl radicals have been proposed as possible novel bactericidal antibiotics^6^. Reactive oxygen species (ROS) are heterogeneous chemicals that include hydroxyl radicals, superoxide anions, and non-radical species such as hydrogen peroxide. ROS are metabolic byproducts produced endogenously by bacteria and can lead to DNA, RNA, proteins and lipids damage when ROS concentrations exceed critical levels^7^. *Mtb* uses catalase-peroxidase (KatG) as the first line of defence against an excess of ROS. Our preliminary study showed that the anti-*Mtb* effect of some aminothiourea derivatives was associated with inhibition of KatG activity^8^^,^^9^. These findings suggested that *Mtb* KatG may be used as a novel target for developing anti-*Mtb* agents.

In the last two decades, some of the 1,2,4-triazole derivatives have been found to have strong activity against *Mtb*^10^^,^^11^. In the present study, we describe the synthesis and the *in vitro* anti-*Mtb* activity of fifteen 1,2,4-triazole derivatives (compounds **1**–**15**). In addition, the mechanism of action of compound **4** was initially predicted by molecular docking, enzyme inhibition assay, ROS measurement, and cell image analysis.

## Results and discussion

### Chemistry

**Scheme 1.**
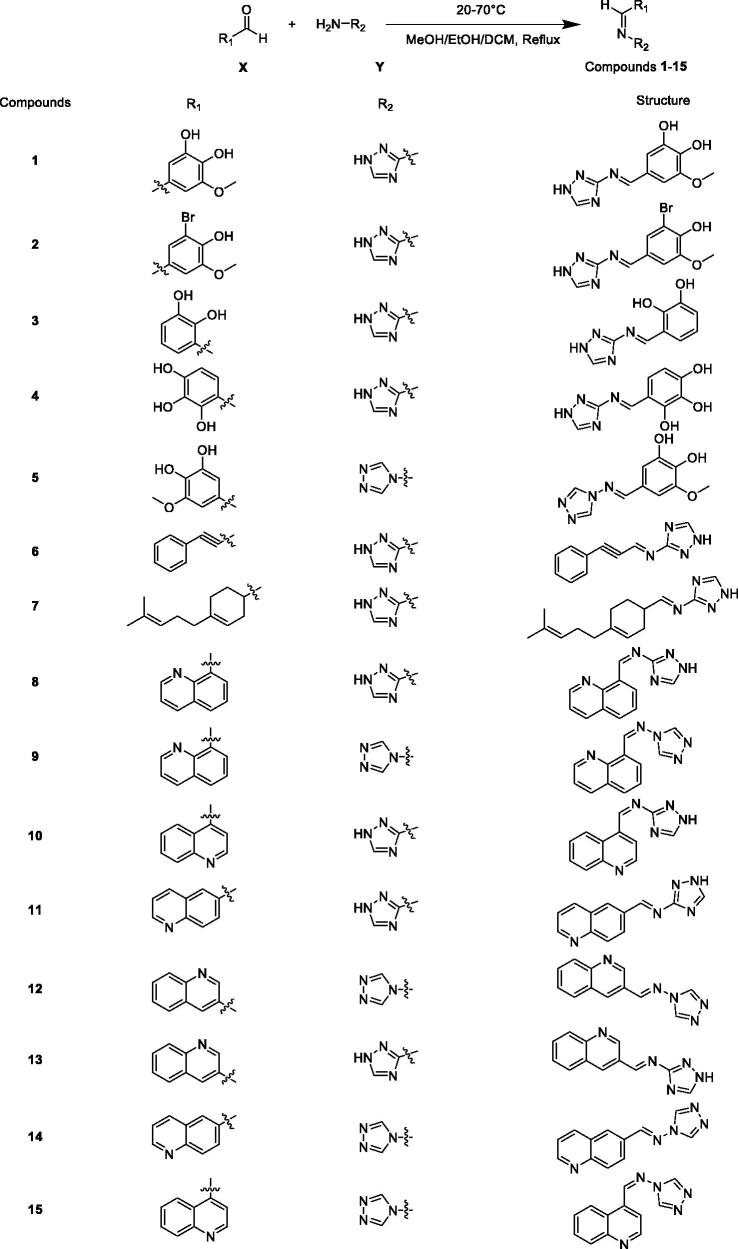
Synthetic routes of the compounds **1**–**15**.

The synthesis of the compounds **1**–**15** was achieved according to the steps illustrated in Scheme 1. The yield of most derivatives is around 60% and the methods employed are very simple. Compounds **1**–**5** were synthesised in the presence of a catalytic amount of glacial acetic acid. The difference is that compounds **11**–**15** were synthesised in the presence of catalytic amounts of hydrochloric acid. On the whole, the synthesis of these compounds is uncomplicated and the products are easy to purify and therefore inexpensive. Currently, TB occurs mainly in poor populations in developing countries. Drug-resistant TB, in particular, takes a long time to treat and the drugs are expensive, making treatment unaffordable for most patients. Therefore, compounds with complex synthetic routes were generally not considered in the design of TB drugs in this study.

### Antibacterial activity

As shown in Table 1, all 15 compounds exhibited activities against *Mtb*. Among them, compound **4** was the most potent compound against *Mtb*. Overall, compounds with high scoring values for docking with *Mtb* KatG showed stronger activity against *Mtb*, with MIC values ranging from 2 to 16 μg/mL for compounds (**2**, **3**, **4**, **5**, **6**, **7**, **10**, **11**, **12**, and **13**) with scoring values more than 110, except for compound **1**. Conversely, compounds (**8**, **9**, **14**, and **15**) with scoring values below 95 had weaker anti-*Mtb* activity, all with MIC values of 32 μg/mL. Isoniazid (INH), after activated by the catalase-peroxidase KatG, can inhibit InhA, the enoyl reductase from *Mtb*, by forming a covalent adduct with the NAD cofactor^12^. Since KatG is not the anti-*Mtb* target of INH, although the anti-*Mtb* activity of INH is strong, the molecular docking score of INH and KatG is not high.

**Table 1.** Anti-*Mtb* activity, molecular docking score, and log *P* of the compounds **1**–**15**.

Compounds	MIC (μg/mL)	Docking score	Log *P*^a^
**1**	32	124.4	0.29
**2**	16	115.4	1.55
**3**	8	115.5	0.48
**4**	2	135.9	0.21
**5**	4	135.1	−0.06
**6**	16	112.9	1.48
**7**	16	118.2	3.61
**8**	32	93.9	1.20
**9**	32	67.1	0.40
**10**	16	130.8	1.48
**11**	8	123.8	1.23
**12**	16	128.1	0.88
**13**	16	123.5	1.23
**14**	32	80.0	0.88
**15**	32	86.3	1.13
Isoniazid	0.25	99.9	−0.64
Pyrazinamide	120	85.3	−1.31

^a^ Predicted by ChemDraw.

Log *P* values, also known as octanol–water partition coefficients, is used as an indicator of the lipophilicity and solubility of a compound, and to predict its potential for entry into cells. The cell wall of *Mtb* contains a large amount of mycolic acid. Theoretically, the greater the Log *P* values of the compound, the easier it is to enter the *Mtb* cells and potentially produce a stronger antibacterial effect^13^. The Log *P* values of the 15 compounds are shown in Table 1. Compound **4**, the compound with the strongest anti-*Mtb* effect in this experiment, had a Log *P* values of 0.21. The Log *P* values (0.29) of compound **1** were close to that of compound **4**, but it had a weaker anti-*Mtb* effect. Despite the high Log *P* values of 3.61 for compound **7**, it did not have a prominent antibacterial effect. Overall, the anti-*Mtb* effects of these compounds lacked correlation with their Log *P* values.

### Time-kill curve

**Figure 1.**
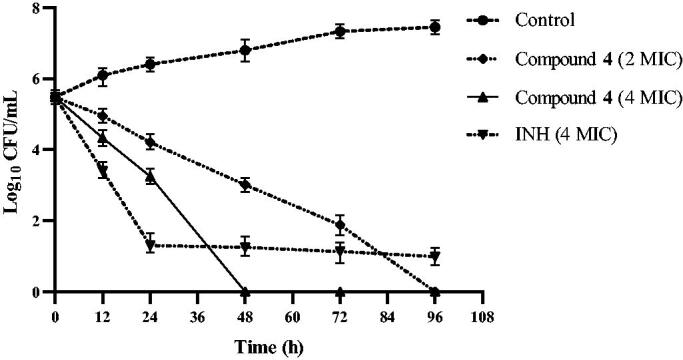
Time-killing curve of compound **4**.

Compound **4** has a strong bactericidal effect as shown in Figure 1. At a concentration of 4 MIC (8 μg/mL), it killed all *Mtb* within 48 h. After the concentration of compound **4** was reduced to 2 MIC (4 μg/mL), its bactericidal effect decreased slightly, but all *Mtb* was still killed by it within 96 h. The results suggest that compound **4** is both a time-dependent and a concentration-dependent bactericidal agent against *Mtb*. INH at a concentration of 4 MIC (1 μg/mL) kills most *Mtb* within 24 h. This result is consistent with other reports^14^. It was obvious that INH failed to kill a very small subpopulation of phenotypically drug-resistant *Mtb* even after 96 h. These drug-resistant *Mtb* subpopulations are the greater risk of relapse of TB^15^. This may partially explain the long treatment time currently required to treat TB. In this study, time-kill experiments were performed in static systems, and drug concentrations were constant without media exchange. It is necessary to evaluate the bactericidal effect of compound **4** using the *in vivo* time-kill method. Nevertheless, the bactericidal effect of compound **4** provides an opportunity for the development of new drugs to shorten the duration of TB treatment.

### *Mtb* morphology

**Figure 2.**
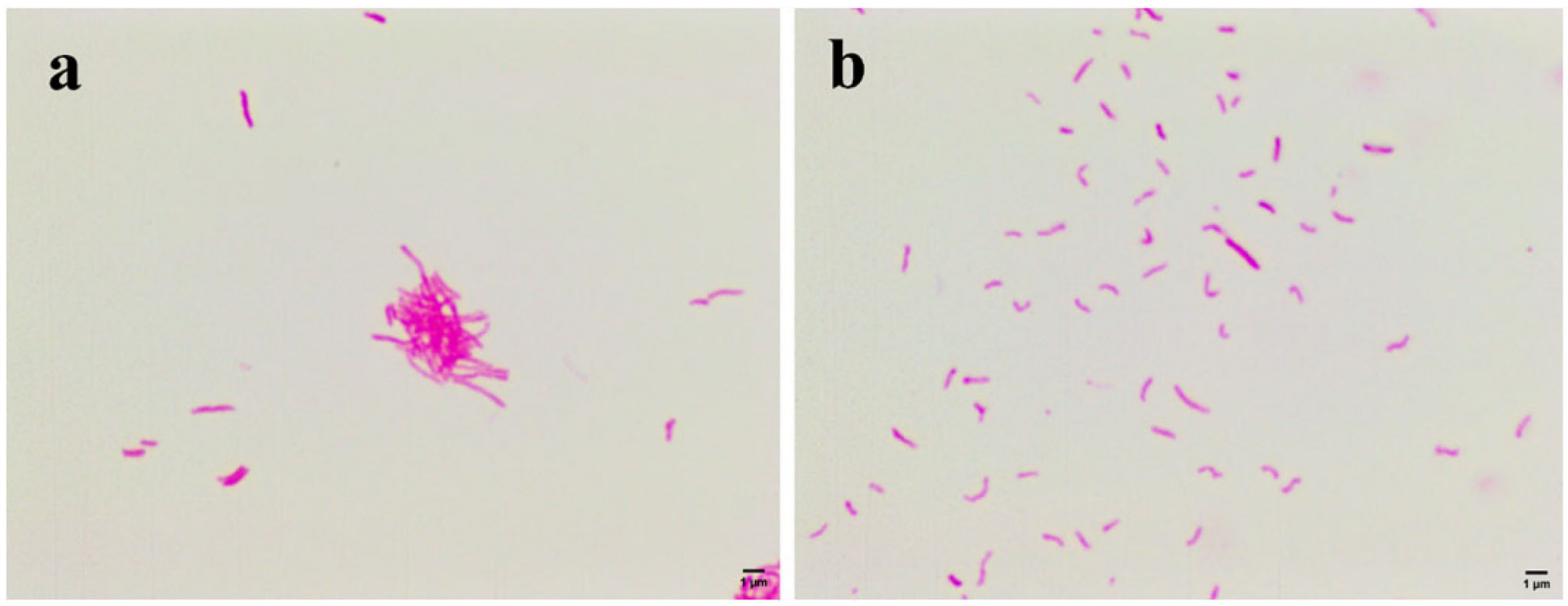
Morphological changes of *Mtb.* (a) Negative control group; (b) treatment with 1 μg/mL compound **4**.

Figure 2 shows the morphological changes of *Mtb* treated with subinhibitory concentrations (1 μg/mL) of compound **4**. Compared to the negative control (Figure 2(a)), *Mtb* became small and curved and lighter in colouration after treatment with compound **4** (Figure 2(b)).

*Mtb* is difficult to stain because their lipid capsules are in the cell wall. However, once the lipid capsules are stained with carbocyanin, the newly formed compound will resist decolourisation by acid alcohol and retain the original staining colour (red). Therefore, after the Ziehl-Neelsen stain, the integrity of the cell wall of *Mtb* can be judged by the shade of red colour of the bacilli. The fading of the red colour indicates that the lipids of the cell wall of *Mtb* are damaged such as oxidative damage^16^. In addition, the shape of *Mtb* is determined by the cell wall. For this reason, we speculate that compound **4** has a disruptive effect on the cell wall of *Mtb*.

### Inhibition of KatG enzyme

*Mtb* will produce ROS as a by-product during normal metabolism. ROS are capable of damaging DNA, RNA, proteins, and lipids, leading to cell death when ROS levels exceed the detoxification and repair capacity of *Mtb.* However, *Mtb* can reduce the more toxic hydrogen peroxide to water and molecular oxygen by KatG enzyme. Inhibition of *Mtb* KatG will result in increased levels of endogenous ROS. Therefore, KatG may become a target for developing novel anti-*Mtb* drugs^7^^,^^17^.

**Figure 3.**
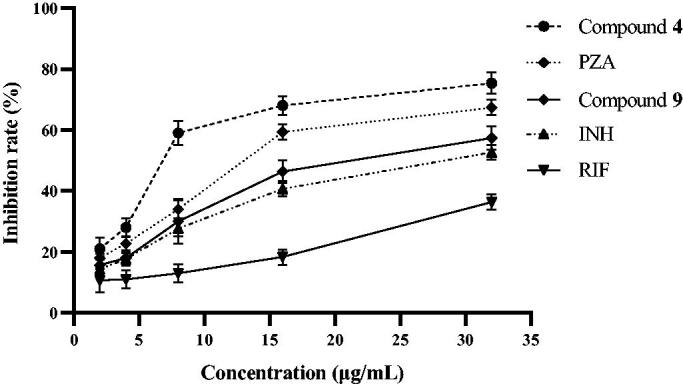
Inhibition of *Mtb* KatG by compounds **4** and **9**.

Table 1 shows that among the 15 compounds, compound **4** had the greatest affinity for KatG, while compound **9** had a poor affinity for KatG. To verify the molecular docking results, the inhibitory activities of compounds **4** and **9** against *Mtb* KatG were determined in this experiment. Figure 3 shows that compounds **4** and **9** exhibited inhibitory activity against KatG of *Mtb* in a dose-dependent manner. However, compound **4** had a stronger inhibitory activity against KatG than **9**. For example, at a concentration of 8 μg/mL, the inhibiting rate of KatG by compound **4** was 59%, while that of compound **9** was only 30%. The docking simulation results are consistent with the actual measured values. Therefore, KatG may be the target of these compounds against *Mtb*. Pyrazinamide (PZA) is a first line anti-tubercular drug for which the mechanism of action remains unresolved^18^. Interestingly, PZA also has the ability to inhibit the KatG of *Mtb*. Could the antibacterial mechanism of PZA be related to the inhibition of KatG? In the next work, we intend to investigate it in depth.

### ROS measurement

**Figure 4.**
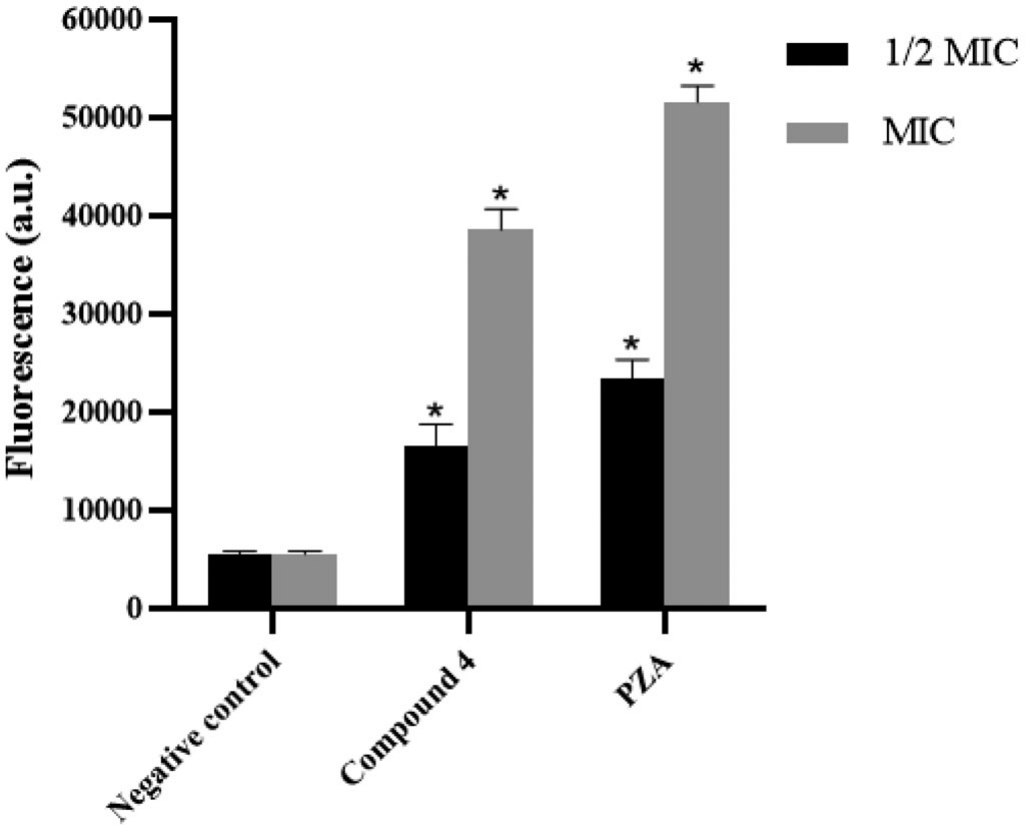
Intracellular ROS production of *Mtb* that treated with compound **4** and PZA.

ROS consist of radical oxygen species including superoxide anion (O_2_•−) and hydroxyl radical (•OH) and non-radical oxygen species such as hydrogen peroxide (H_2_O_2_). The fluorescent probe 2′,7′-dichlorofluorescin diacetate (DCFH-DA) was used to measure ROS in *Mtb* cells, where fluorescence intensity is positively correlated with the amount of ROS in the cells. Figure 4 shows that compound **4** could significantly increase the level of ROS in *Mtb* cells at 1/2 MIC (1 μg/mL) and MIC (2 μg/mL) concentrations. Elevated ROS levels presumably associated with inhibition of *Mtb* KatG by compound **4**. Accordingly, because PZA could inhibit KatG enzyme, it also caused intracellular ROS accumulation in *Mtb*.

### Cytotoxicity

The results of the cytotoxicity assay are shown in Table 2. The IC_50_ value was defined as the highest drug concentration at which 50% of the cells are viable. Selectivity index (SI) is expressed by the IC_50_/MIC ratio^19^. The SI is ≥10, suggesting that the compound is amenable to further study^20^. The SI of compound **4** is greater than 10, indicating that the compound has low toxicity to *Vero* cells and has the potential to become a drug.

**Table 2.** Cytotoxicity on *Vero* cells of compound **4**.

Compound	IC_50_ (μg/mL)	SI
**4**	126.23 ± 2.54	31.56
INH	72.88 ± 1.23	291.2

### Molecular docking

**Figure 5.**
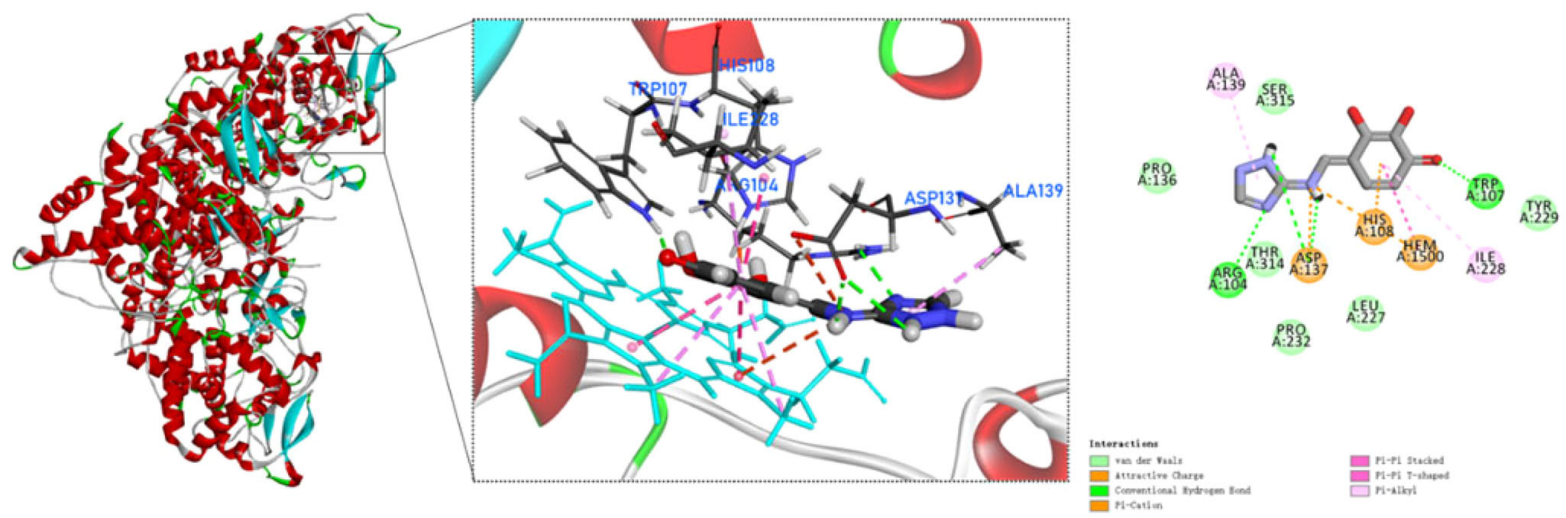
Molecular docking analysis for compound **4**.

To date, none of the crystal structures of *Mtb* KatG contains small molecule inhibitors that co-crystallise with the enzyme protein. However, the active site of *Mtb* KatG contained a heme protoporphyrin IX moiety^17^. It provided us with assistance in selecting the active site for KatG when performing docking simulations. To explore the binding mode and interaction of compound **4** with the active site of *Mtb* KatG (PDB ID: 1SJ2), molecular docking studies were performed by Discovery Studio software. Figure 5 shows the docking interactions between compound **4** with KatG. There is pi–pi stacked interactions between compound **4** and KatG heme. It indicates that the binding site of compound **4** to KatG is the active site. In addition, compound **4** is interacting with six amino acids residues of KatG and forms four hydrogen bonds. This may explain why compound **4** has the highest score for molecular docking with KatG. *Mtb* KatG contains an adduct consisting of Trp107, Tyr229, and Met255 in its distal heme pocket. This covalent adduct plays an important role in KatG of wild type *Mtb* to ensure high catalytic activity^21^. A hydroxyl group on the benzene ring of compound **4** forms a hydrogen bond with Trp107, which may change the geometry of the adduct and lead to a decrease in the catalytic activity of KatG.

**Figure 6.**
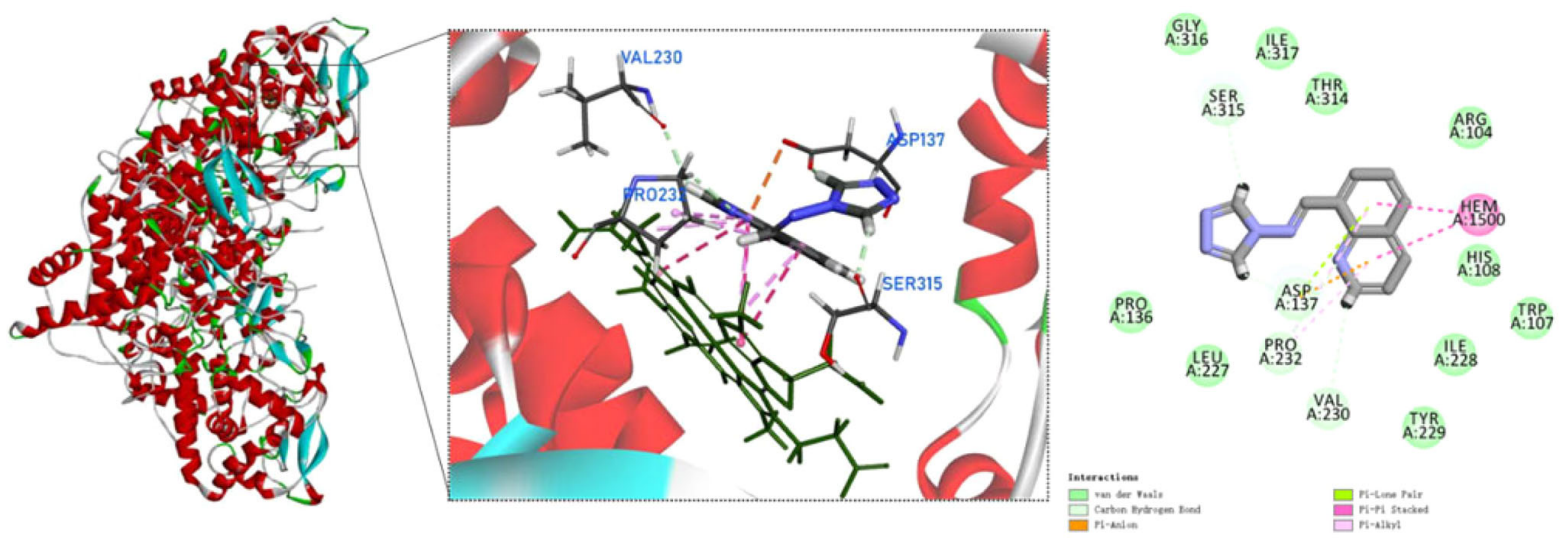
Molecular docking analysis for compound **9**.

Compound **9** also has a pi–pi stacked interactions with the heme of KatG, but fails to form hydrogen bonds with amino acid residues in the active site of KatG and fails to produce any binding to the adduct of Met^255^–Tyr^229^–Trp^107^ (Figure 6). Therefore, compound **9** has a low scoring value with KatG. The experimental test results are in agreement with the molecular docking simulations.

Significantly, the anti-*Mtb* activities of the 15 compounds were in general agreement with their molecular docking scoring values (Table 1). Among these compounds, compound **4** firmly binds to the KatG active site, exhibits strong KatG enzyme inhibitory activity, causes elevated intracellular ROS in *Mtb*, alters *Mtb* morphology and stainability, and has potent bactericidal effects. These results suggest that *Mtb* KatG is a likely antimycobacterial target of compound **4**.

## Conclusions

In this study, fifteen 1,2,4-triazole derivatives were synthesised and evaluated for their antimycobacterial activity. Among the 15 compounds, compound **4** showed the strongest anti-*Mtb* activity with an MIC of 2 μg/mL. Molecule docking exhibited that compound **4** had a good affinity for *Mtb* KatG. The experiment indicated that compound **4** had inhibitory activity against KatG enzyme in a dose–response manner. In addition, *Mtb* showed intracellular accumulation of ROS after treatment with compound **4**. Compound **4** caused shape changes and staining changes in *Mtb* at subinhibitory concentrations. These may be due to oxidative damage of cell wall components by ROS. Therefore, it can be concluded that the anti-*Mtb* effect of compound **4** may be related to its inhibition of KatG enzyme. This study provides a new idea for the development of novel anti-*Mtb* drugs.

## Materials and methods

### General

All materials and reagents used for synthesis and analysis were commercially available and were of analytical grade and used without further purification. All reactions were monitored by thin layer chromatography (TLC) using pre-coated silica gel GF 254 plate (Yantai Wish Chemical Products Co., Ltd., Yantai, China). Column chromatography was performed using silica gel (Qingdao Marine Chemical Inc., Qingdao, China). The ^1^H and ^13^C NMR spectra of the compounds were recorded using a Bruker ACF-400, 400 MHz spectrometer (Bruker Bioscience, Billerica, MA) with tetramethylsilane as an internal standard and DMSO-*d*_6_ as solvent, with chemical shifts reported in *δ* values.

### General procedure for the synthesis of compounds 1–15

In this experiment, 15 compounds belong to Schiff base compounds, which are formed by condensation reaction of aldehydes and amines. As shown in Scheme 1, the material **X** was substituted aromatic aldehydes or quinoline aldehydes, and the material **Y** was aminotriazoles. Using methanol or ethanol or dichloromethane as the solvent, **X** and **Y** were mixed and stirred at 20–70 °C under reflux. The reaction time is typically 6–12 h to give compounds **1**–**15**. The detailed methods for the synthesis of these 15 compounds are described as follows.

#### 5-(((1H-1,2,4-triazol-3-yl)imino)methyl)-3-methoxybenzene-1,2-diol (1)

3,4-Dihydroxy-5-methoxybenzaldehyde (168.15 mg, 1 equiv.) was added to 6 mL of absolute ethanol, and after complete dissolution, 1H-1,2,4-triazole-3-amine (100.90 mg, 1.2 equiv.) was added, and a catalytic amount of glacial acetic acid (68.63 μL, 1.2 equiv.) was added to the reaction system. The mixed solution was stirred at room temperature for 9 h. The reaction was monitored by TLC and finally a light green precipitate was obtained. The precipitate was washed with anhydrous ethanol and then filtered to obtain compound **1**.

Light green powder (yield: 126.4 mg, 54%). ^1^H NMR (400 MHz, DMSO-*d*_6_) *δ* 13.92 (s, 1H), 9.32 (s, 1H), 8.98 (s, 1H), 7.14 (s, 1H), 7.10 (s, 1H), 3.83 (s, 3H). ^13^C NMR (101 MHz, DMSO-*d*_6_) *δ* 164.15, 148.50, 145.91, 141.10, 139.26, 127.35, 125.70, 110.70, 104.75, 55.94.

#### 4-(((1H-1,2,4-triazol-3-yl)imino)methyl)-2-bromo-6-methoxyphenol (2)

3-Bromo-4-hydroxy-5-methoxybenzaldehyde (231.04 mg, 1 equiv.) was added to 7 mL of absolute ethanol, and after complete dissolution, 1H-1,2,4-triazol-3-amine (100.90 mg, 1.2 equiv.) was added, and a catalytic amount of glacial acetic acid (68.63 μL, 1.2 equiv.) was added to the reaction system. The mixed solution was stirred at room temperature for 8 h. The reaction was monitored by TLC and finally a yellow-green precipitate was obtained. The precipitate was washed with anhydrous ethanol and then filtered to obtain compound **2**.

Yellow-green powder (yield: 161.1 mg, 54.2%). ^1^H NMR (400 MHz, DMSO-*d*_6_) *δ* 13.96 (s, 1H), 10.37 (s, 1H), 9.07 (s, 1H), 8.49 (s, 1H), 7.75 (s, 1H), 7.60 (s, 1H), 3.92 (s, 3H). ^13^C NMR (101 MHz, DMSO-*d*_6_) *δ* 161.40, 149.45, 148.20, 128.48, 128.34, 127.12, 109.30, 109.16, 108.92, 55.86.

#### 3-(((1H-1,2,4-triazol-3-yl)imino)methyl)benzene-1,2-diol (3)

2,3-Dihydroxybenzaldehyde (138.12 mg, 1 equiv.) was added to 5 mL of absolute ethanol, and after complete dissolution, 1H-1,2,4-triazole-3-amine (100.90 mg, 1.2 equiv.) was added, and a catalytic amount of glacial acetic acid (68.63 μL, 1.2 equiv.) was added to the reaction system. The mixed solution was stirred at room temperature for 12 h. The reaction was monitored by TLC and finally an orange-yellow precipitate was obtained. The precipitate was washed with anhydrous ethanol and then filtered to obtain compound **3**.

Orange-yellow powder (yield: 108.8 mg, 53.3%). ^1^H NMR (400 MHz, DMSO-*d*_6_) *δ* 14.12 (s, 1H), 12.57 (s, 1H), 9.37 (s, 2H), 8.58 (s, 1H), 7.21 (d, *J* = 6.5 Hz, 1H), 6.99 (d, *J* = 7.7 Hz, 1H), 6.85–6.75 (m, 1H). ^13^C NMR (101 MHz, DMSO-*d*_6_) *δ* 165.62, 164.12, 149.29, 145.71, 144.37, 122.92, 121.31, 119.73, 119.27.

#### 4-(((1H-1,2,4-triazol-3-yl)imino)methyl)benzene-1,2,3-triol (4)

2,3,4-Trihydroxybenzaldehyde (154.12 mg, 1 equiv.) was added to 5 mL of absolute ethanol, and after complete dissolution, 1H-1,2,4-triazole-3-amine (100.90 mg, 1.2 equiv.) was added, and a catalytic amount of glacial acetic acid (68.63 μL, 1.2 equiv.) was added to the reaction system. The mixed solution was stirred at room temperature for 12 h. The reaction was monitored by TLC and finally a yellow precipitate was obtained. The precipitate was washed with anhydrous ethanol and then filtered to obtain compound **4**.

Yellow powder (yield: 114.6 mg, 52%). ^1^H NMR (400 MHz, DMSO-*d*_6_) *δ* 13.98 (s, 1H), 12.93 (s, 1H), 9.20 (s, 1H), 8.39 (s, 1H), 7.44 (s, 1H), 7.07 (d, *J* = 8.4 Hz, 1H), 6.47 (d, *J* = 8.5 Hz, 1H), 5.80 (s, 1H). ^13^C NMR (101 MHz, DMSO-*d*_6_) *δ* 165.57, 153.36, 151.47, 150.92, 132.44, 132.28, 124.89, 112.03, 108.33.

#### 5-(((4H-1,2,4-triazol-4-yl)imino)methyl)-3-methoxybenzene-1,2-diol (5)

3,4-Dihydroxy-5-methoxybenzaldehyde (168.15 mg, 1 equiv.) was added to 5 mL of absolute ethanol, and after complete dissolution, 4H-1,2,4-triazole-4-amine (100.90 mg, 1.2 equiv.) was added, and a catalytic amount of glacial acetic acid (68.63 μL, 1.2 equiv.) was added to the reaction system. The mixed solution was stirred at room temperature for 10 h. The reaction was monitored by TLC and finally a grey precipitate was obtained. The precipitate was washed with anhydrous ethanol and then filtered to obtain compound **5.**

Grey powder (yield: 157.9 mg, 67.4%). ^1^H NMR (400 MHz, DMSO-*d*_6_) *δ* 9.07 (s, 2H), 8.84 (s, 1H), 6.97 (q, *J* = 1.9 Hz, 2H), 3.82 (s, 3H). ^13^C NMR (101 MHz, DMSO-*d*_6_) *δ* 158.51, 148.55, 145.94, 138.88, 138.86, 122.18, 110.25, 103.38, 55.89.

#### 3-Phenyl-N-(1H-1,2,4-triazol-3-yl)prop-2-yn-1-imine (6)

3-Phenylpropiolaldehyde (168.15 mg, 1 equiv.) was dissolved in 10 mL of dichloromethane, and then 1H-1,2,4-triazol-3-amine (100.90 mg, 1.2 equiv.) was added to the reaction system. The mixed solution was stirred at room temperature for 11 h. The reaction was monitored by TLC and finally a light pink precipitate was obtained. The precipitate was washed with dichloromethane and then filtered to obtain compound **6**.

Light pink powder (yield: 118.6 mg, 60.4%). ^1^H NMR (400 MHz, DMSO-*d*_6_) *δ* 14.16 (s, 1H), 11.91 (s, 1H), 8.61 (s, 1H),7.63 (d, *J* = 7.1 Hz, 2H), 7.47 (d, *J* = 7.2 Hz, 2H), 7.34 (s, 1H). ^13^C NMR (101 MHz, DMSO-*d*_6_) *δ* 149.08, 132.21, 131.45, 131.41, 131.25, 130.54, 128.96, 128.66, 128.57, 120.15, 88.33.

#### 1-(4-(4-Methylpent-3-en-1-yl)cyclohex-3-en-1-yl)-N-(1H-1,2,4-triazol-3yl)methanimine (7)

4-(4-methylpent-3-en-1-yl)cyclohex-3-ene-1-carbaldehyde (192.30 mg, 1 equiv.) was dissolved in 6 mL of absolute ethanol, and then 1H-1,2,4-triazol-3-amine (100.90 mg, 1.2 equiv.) was refluxed to the reaction system. The reaction was refluxed at 68.5 °C for 12 h. The reaction was monitored by TLC and finally a white precipitate was obtained. The precipitate was washed with anhydrous ethanol and then filtered to obtain compound **7**.

White powder (yield: 132.3 mg, 51.2%). ^1^H NMR (400 MHz, Chloroform-*d*) *δ* 8.61 (s, 1H), 7.92 (s, 1H), 5.39 (s, 1H), 5.16–4.96 (m, 1H), 2.72–1.65 (m, 11H), 1.65–1.58 (m, 3H), 1.58–1.54 (m, 3H). ^13^C NMR (101 MHz, Chloroform-*d*) *δ* 163.30, 143.43, 138.37, 131.52, 124.38, 119.60, 118.42, 37.63, 28.84, 26.68, 26.39, 25.82, 17.90, 17.84, 17.79.

#### 1-(Quinolin-8-yl)-N-(1H-1,2,4-triazol-3-yl)methanimine (8)

Quinolin-8-formaldehyde (78.6 mg, 1 equiv.) was dissolved in methanol (10 mL), and after being fully dissolved, 1H-1,2,4-triazole-3-amine (84.1 mg, 2 equiv.) was added and mixed. The reaction was carried out at room temperature for 6 h. The reaction was monitored by TLC, and the reaction liquid was dried and purified by methyl-tert-butyl ether. Compound **8** was obtained after filtration.

Yellow powder (yield: 70.1 mg, 63%). ^1^H NMR (400 MHz, DMSO-*d*_6_) *δ* 14.11 (s, 1H), 10.54 (s, 1H), 9.07 (dd, *J* = 4.2, 1.8 Hz, 1H), 8.62 (dd, *J* = 7.3, 1.5 Hz, 1H), 8.51 (dd, *J* = 8.4, 1.9 Hz, 1H), 8.27 (d, *J* = 7.4 Hz, 1H), 7.81 (t, *J* = 7.7 Hz, 1H), 7.67 (dd, *J* = 8.4, 4.2 Hz, 1H). ^13^C NMR (DMSO-*d*_6_, 100 MHz) *δ* 151.13, 150.33, 150.22, 146.41, 144.64, 136.82, 128.57, 128.10, 127.55, 126.61, 121.73.

#### 1-(Quinolin-8-yl)-N-(4H-1,2,4-triazol-4-yl)methanimine (9)

Quinoline-8-formaldehyde (78.6 mg, 1 equiv.) was dissolved in methanol (10 mL), and then mixed with 4H-1,2,4-triazole-4-amine (84.1 mg, 2 equiv.), reacted at 60 °C for 10 h. The reaction was monitored by TLC, and the reaction liquid was dried and purified with ethyl acetate. Compound **9** was obtained after filtration.

Pale brown powder (yield: 90.2 mg, 80.7%). ^1^H NMR (400 MHz, DMSO-*d*_6_) *δ* 10.07 (s, 1H), 9.31 (s, 2H), 9.07 (dd, *J* = 4.2, 1.8 Hz, 1H), 8.53 (dd, *J* = 8.3, 1.8 Hz, 1H), 8.46 (dd, *J* = 7.3, 1.4 Hz, 1H), 8.27 (dd, *J* = 8.2, 1.5 Hz, 1H), 7.79 (dd, *J* = 8.1, 7.4 Hz, 1H), 7.70 (dd, *J* = 8.3, 4.2 Hz, 1H). ^13^C NMR (101 MHz, DMSO-*d*_6_) *δ* 154.55, 151.12, 145.73, 139.18, 136.89, 132.60, 129.10, 128.04, 127.52, 126.59, 122.39.

#### 1-(Quinolin-4-yl)-N-(1H-1,2,4-triazol-3-yl)methanimine (10)

4-Quinoline formaldehyde (78.6 mg, 1 equiv.) was dissolved in methanol (10 mL), which was fully dissolved and mixed with 1H-1,2,4-triazole-3-amine (84.1 mg, 2 equiv.), and reacted at 65 °C for 12 h. The reaction was monitored by TLC, and the reaction liquid was purified with water and ethyl acetate successively. Compound **10** was obtained after filtration.

Grey powder (yield: 64.2 mg, 57.5%). ^1^H NMR (400 MHz, DMSO-*d*_6_) *δ* 9.83 (s, 1H), 9.11–9.08 (m, 2H), 8.51 (s, 1H), 8.16 (d, *J* = 8.5 Hz, 1H), 8.11 (d, *J* = 4.6 Hz, 1H), 7.88 (t, *J* = 7.7 Hz, 1H), 7.78 (t, *J* = 8.0 Hz, 1H). ^13^C NMR (101 MHz, DMSO-*d*_6_) *δ* 162.17, 150.70, 148.56, 137.43, 129.88, 129.83, 128.18, 124.98, 124.60, 122.86.

#### 1-(Quinolin-5-yl)-N-(1H-1,2,4-triazol-3-yl)methanimine (11)

The 6-quinoline formaldehyde (78.6 mg, 1 equiv.) was dissolved in methanol (10 mL), and after being fully dissolved, 1H-1,2,4-triazole-3-amine (84.1 mg, 2 equiv.) was added into the mixture, and then appropriate amount of HCl was added to react at 60 °C for 12 h. The reaction was monitored by TLC, and the reaction liquid was dried. Compound **11** was obtained after purification by ethyl acetate beating and filtration.

Grey powder (yield: 68.4 mg, 61.3%). ^1^H NMR (400 MHz, DMSO-*d*_6_) *δ* 14.13 (s, 1H), 9.41 (s, 1H), 9.00 (s, 1H), 8.60 (s, 1H), 8.52 (d, *J* = 8.3 Hz, 1H), 8.40 (d, *J* = 8.3 Hz, 1H), 8.13 (d, *J* = 8.6 Hz, 1H), 7.64 (s, 1H). ^13^C NMR (101 MHz, DMSO-*d*_6_) *δ* 152.31, 149.44, 137.10, 129.85, 127.77, 127.12, 122.39.

#### 1-(Quinolin-3-yl)-N-(4H-1,2,4-triazol-4-yl)methanimine (12)

The 3-quinoline formaldehyde (78.6 mg, 1 equiv.) was dissolved in anhydrous ethanol (10 mL). After being fully dissolved, 4H-1,2,4-triazole-4-amine (84.1 mg, 2 equiv.) was added to the mixture, and then an appropriate amount of HCl was added to react. The mixed solution was stirred at room temperature for 10 h. The reaction was monitored by TLC and finally a white precipitate was obtained, and the precipitate was washed with anhydrous ethanol and then filtered to obtain compound **12**.

White powder (yield: 64.2 mg, 57.5%). ^1^H NMR (400 MHz, DMSO-*d*_6_) *δ* 9.35 (d, *J* = 2.2 Hz, 1H), 9.33 (s, 1H), 9.23 (s, 2H), 8.79 (d, *J* = 2.1 Hz, 1H), 8.20 (dd, *J* = 8.1, 1.5 Hz, 1H), 8.12 (dd, *J* = 8.5, 1.1 Hz, 1H), 7.91 (ddd, *J* = 8.4, 6.9, 1.5 Hz, 1H), 7.74 (ddd, *J* = 8.1, 6.9, 1.2 Hz, 1H). ^13^C NMR (101 MHz, DMSO-*d*_6_) *δ* 156.15, 148.71, 148.43, 137.29, 131.73, 129.25, 128.97, 127.80, 126.95, 125.43.

#### 1-(Quinolin-3-yl)-N-(1H-1,2,4-triazol-3-yl)methanimine (13)

The 3-quinoline formaldehyde (78.6 mg, 1 equiv.) was dissolved in methanol (10 mL), which was fully dissolved, mixed with 1H-1,2,4-triazole-3-amine (84.1 mg, 2 equiv.), and then added with the appropriate amount of HCl. The mixed solution was stirred at room temperature for 12 h. The reaction was monitored by TLC and finally a white precipitate was obtained, and the precipitate was purified by methyl tert-butyl ether and filtered to obtain compound **13**.

White powder (yield: 68.1 mg, 61%). ^1^H NMR (400 MHz, DMSO-*d*_6_) *δ* 14.16 (s, 1H), 9.50 (d, *J* = 2.1 Hz, 1H), 9.46 (s, 1H), 8.96 (d, *J* = 2.1 Hz, 1H), 8.45 (s, 1H), 8.14 (ddd, *J* = 15.6, 8.3, 1.2 Hz, 2H), 7.90 (ddd, *J* = 8.4, 6.9, 1.5 Hz, 1H), 7.72 (ddd, *J* = 8.1, 6.8, 1.2 Hz, 1H). ^13^C NMR (101 MHz, DMSO-*d*_6_) *δ* 149.34, 148.88, 138.50, 131.55, 129.28, 128.96, 128.19, 127.59, 127.14.

#### 1-(Quinolin-5-yl)-N-(4H-1,2,4-triazol-4-yl)methanimine (14)

The 6-quinoline formaldehyde (78.6 mg, 1 equiv.) was dissolved in methanol (10 mL), and after being fully dissolved, 4H-1,2,4-triazole-4-amine (84.1 mg, 2 equiv.) was added into the mixture, and the appropriate amount of HCl was added. The mixed solution was reacted at room temperature for 10 h. The reaction response was monitored by TLC and finally a white precipitate was obtained. Compound **14** was obtained after filtration.

White powder (yield: 52.7 mg, 47.2%). ^1^H NMR (400 MHz, DMSO-*d*_6_) *δ* 9.28 (s, 1H), 9.21 (s, 2H), 9.01 (dd, *J* = 4.3, 1.7 Hz, 1H), 8.60–8.53 (m, 1H), 8.40 (d, *J* = 1.9 Hz, 1H), 8.25 (dd, *J* = 8.9, 1.9 Hz, 1H), 8.15 (d, *J* = 8.8 Hz, 1H), 7.65 (dd, *J* = 8.3, 4.2 Hz, 1H). ^13^C NMR (101 MHz, DMSO-*d*_6_) *δ* 157.37, 152.42, 149.16, 139.07, 137.05, 131.30, 130.32, 130.15, 127.69, 126.43, 122.53.

#### 1-(Quinolin-4-yl)-N-(4H-1,2,4-triazol-4-yl)methanimine (15)

4-Quinoline formaldehyde (78.6 mg, 1 equiv.) was dissolved in methanol (10 mL), and then mixed with 4H-1,2,4-triazole-4-amine (84.1 mg, 2 equiv.). An appropriate amount of HCl was added, and the reaction was stirred at room temperature for 12 h. The reaction response was monitored by TLC and finally a yellow precipitate was obtained. Compound **15** was obtained after filtration.

Yellow powder (yield: 73.6 mg, 65.9%). ^1^H NMR (400 MHz, DMSO-*d*_6_) *δ* 9.77 (s, 1H), 9.36 (s, 2H), 9.11 (d, *J* = 4.4 Hz, 1H), 8.83 (dd, *J* = 8.6, 1.3 Hz, 1H), 8.17 (dd, *J* = 8.4, 1.4 Hz, 1H), 8.01 (d, *J* = 4.5 Hz, 1H), 7.90 (ddd, *J* = 8.4, 6.9, 1.4 Hz, 1H), 7.80 (ddd, *J* = 8.4, 6.9, 1.4 Hz, 1H). ^13^C NMR (101 MHz, DMSO-*d*_6_) *δ* 155.55, 150.99, 148.90, 139.78, 136.15, 130.60, 130.29, 128.40, 125.36, 125.00, 120.83.

### Anti-*Mtb* assay

The anti-*Mtb* activity of the compounds was determined by resazurin microtitre plate assay (REMA)^22^. *Mtb* H37Rv (ATCC 27294) was used as reference strain, and resazurin used as an oxidation–reduction indicator. The REMA plate method was performed in 7H9 broth containing of 0.1% casitone, 0.5% glycerol, oleic acid, albumin, glucose, and peroxidase (Becton-Dickinson, Sparks, MD). Compounds were added to the 7H9 broth at a final concentration of 1–32 μg/mL in 96-well plates. Isoniazid was used as a positive control. Antibiotic-free growth control and sterility control without inoculation were also included. The inoculum was adjusted to McFarland 1.0 using a turbidimeter from fresh colonies on 7H11 agar, diluted 1:10 in 7H9 broth, with 100 μL as the inoculum. The plates were covered and sealed in plastic bag, and incubated at 37 °C in a normal atmosphere. After seven days of incubation, 30 μL of resazurin solution was added to each well, incubated for 48 h at 37 °C, and colour development was assessed. If the blue colour in the wells turns pink, it indicates that *Mtb* is growing, and MIC is defined as the minimum concentration of the compound that prevents the colour change. The *in vitro* assay was performed in triplicate and repeated at least twice.

### Time-kill curve

Time-kill curve refers to fixing a series of antibacterial drug concentrations and observing the bactericidal activity of the drug on the test bacteria and the process of bactericidal rate change with concentration. It can distinguish whether the drug is a bacteriostatic or bactericidal agent. Among the 15 compounds, compound **4** showed the strongest activity against *Mtb*, therefore, compound **4** was chosen to explore its bactericidal profile. The test was performed as previously reported^23^. Briefly, *Mtb* was treated with compound **4** at concentrations of 2 MIC (4 μg/mL) and 4 MIC (8 μg/mL). Isoniazid (INH) was used as reference drug. Drug-free 7H9 broth was used as negative controls. The inoculum (100 μL) was added into each sample and placed in a 37 °C incubator. Approximately, 200 μL of the medium was collected at time 0 and every 12 h thereafter until the end of the experiment. The number of viable bacteria in each sample was determined by plating serial dilutions of the samples on antibiotic-free Middlebrook 7H11 agar plates. The plates were incubated for 3–4 weeks at 37 °C. The antibacterial activity was measured as the reduction in number of viable bacteria, expressed as CFU/mL. Each experiment was performed in duplicate for each dose level. Time-kill curves were constructed based on the time course of CFU/mL measurements.

### *Mtb* morphology

To initially determine the effect of compound **4** on the morphology and acid-fast stain of *Mtb*, *Mtb* was treated with subinhibitory concentrations (1 μg/mL) of compound **4**, incubated for seven days, and images were collected by light microscopy after Ziehl-Neelsen staining. The drug-free *Mtb* was used as a negative control.

### *In vitro* enzyme inhibition assay

The ultraviolet absorption values of H_2_O_2_ were determined by UV spectrophotometry^24^. The measurement wavelength was set at 240 nm, and the ambient temperature was controlled between 24 and 26 °C during the test. The KatG enzyme was isolated and purified from *Mtb* H37Rv according to the literature^8^^,^^25^. In this assay, the KatG solution (1 mg/mL) was prepared with phosphate-buffered saline (PBS, 0.01 M, pH 7.4). The activity unit of above enzyme solution is 10 000 U/mL. Reference drugs (isoniazid, PZA, and rifampin) and compound **4** were diluted with PBS into 2, 4, 8, 16, and 32 μg/mL solutions. Hydrogen peroxide (10 mM) was prepared by adding 0.1134 mL of 30% hydrogen peroxide to 100 mL of PBS. The catalytic activity of KatG was determined according to the procedure demonstrated in Table 3. The enzyme inhibition rate was calculated according to the following formula:

$$\text{Inhibition rate }\left(\%\right)\;=\;\left\{1-\left({\text{OD}}_{\text{Standard}}-{\text{ OD}}_{\text{Test}}/{\text{OD}}_{\text{Standard}}\right)\right\}\;\times \text{ 1}00\%.$$

**Table 3.** The procedures for evaluating catalase activity of *Mtb* KatG^8^.

Reagents	Test	Standard	Blank
KatG solution	500 μL	–	–
PBS	–	1500 μL	2500 μL
Samples (INH, RIF, PZA, compounds **4** and **9**)	1000 μL	–	–
Mix the tube with vortex and incubate at 25 °C for 30 min, then add hydrogen peroxide:			
H_2_O_2_	1000 μL	1000 μL	–
Spin the tubes for 10 s and hold at 25 ± 1 °C for 2 min. Then, add 1000 μL of 1.8 M sulphuric acid to stop the reaction. Record the absorbance at the reagent blank at 240 nm.			

### ROS measurement

*Mtb* relies on KatG enzyme to reduce hydrogen peroxide and keep intracellular ROS in relative equilibrium. Inhibition of *Mtb* KatG enzyme prevents hydrogen peroxide from being reduced and will result in the accumulation of ROS in the cells. In the presence of ROS such as superoxide anion radical, hydrogen peroxide, and hydroxyl radical, DCFH-DA (2′,7′-dichlorofluorescin diacetate) is rapidly oxidised to highly fluorescent 2′,7′-dichlorofluorescein (DCF). In this experiment, the final concentration of compound **4** was set at 1/2 MIC (1 μg/mL) and MIC (2 μg/mL). Correspondingly, the final concentration of the reference drug PZA was also set at 1/2 MIC (60 μg/mL) and MIC (120 μg/mL). DCFH-DA fluorescent probe was used to measure intracellular ROS levels^9^. Briefly, mycobacterial cells (1 × 10^10^ CFU/mL) treated with drugs (that untreated used as negative control) were incubated under 37 °C for 4 h, DCFH-DA solution (5 μM) was added to the cultures and reincubated for 30 min. The mycobacterial suspension was then centrifuged, washed, and resuspended in PBS buffer to remove extracellular excess DCFH-DA. The fluorescent signal (*λ*_ex_/*λ*_em_ = 484/525 nm) was measured on a fluorescence Microplate Reader (SpectraMax Gemini EM) to evaluate ROS production in mycobacteria cells.

### *In vitro* cytotoxicity assays

Cytotoxicity of compound **4** was determined with the *Vero* cell line (ATCC CCL-81) using the MTT (3-(4,5-dimethyl-2-thiazolyl)-2,5-diphenyl-2H-tetrazolium bromide) assay as previously described^26^. First, the *Vero* cells at logarithmic growth stage were taken and prepared into a cell suspension with a density of 2 × 10^5^ cells/mL using RPMI-1640 culture medium. The cell suspension was dispensed in 96-well plates at 200 μL per well. Different concentrations of the drug olution were added to the corresponding wells. Isoniazid (INH) was used as reference drug. The negative control group was the cell suspension without drug treatment. The final concentrations of compound **4** and INH were 20, 40, 80, 160, and 320 μg/mL. The 96-well plates were incubated in a CO_2_ incubator for 72 h at a temperature of 37 °C. Then, the supernatant was aspirated and 200 μL of RPMI-1640 medium and 20 μL of MTT were added to each well and incubated for 4 h in a CO_2_ incubator at 37 °C. Finally, centrifuge, remove the supernatant, add 200 μL of DMSO to each well, shake for 15 min, and detect the OD_570_ value of each well. The samples were replicated twice for each concentration in parallel three times. The half maximum inhibitory concentration (IC_50_) is defined as the concentration of the test sample required to inhibit half of *Vero* cell viability. The SI of the test sample is calculated by dividing the IC_50_ value by the MIC value.

### Molecular docking

The crystal structure of *Mtb* KatG (PDB ID: 1SJ2) was obtained from the Protein Data Bank (PDB; https://www.rcsb.org) and prepared as the receptor in Discovery Studio by removing water and ligands, adding hydrogens, and minimising energy. The centre of the active site was set at 43.482, –9.32, and 28.8632 Å for *x*, *y*, and *z* centres, respectively. The grid box was set to 90 Å × 90 Å × 90 Å, and the grid point spacing was 0.5 Å. The 2D structures of the compound **1**–**15** were drawn using ChemDraw, and then energy minimisation was performed using the MM2 module in ChemDraw 3D, followed by saving the files as Mol2 format. The molecular docking was carried out using Discovery Studio Visualizer v.21.1.0 and the results were analysed visually^27^.

### Statistical analysis

Statistical analysis was performed using the Student *t*-test and significance was assigned at *p* < 0.05.

## Supplementary Material

Supplemental Material
