# Supplementary material for: Synthesis, antimycobacterial evaluation, and molecular docking study of 1,2,4-triazole derivatives
Source: J Enzyme Inhib Med Chem. 2023 Jun 29;38(1):2229070. doi: 10.1080/14756366.2023.2229070 (PMC10312031; doi:10.1080/14756366.2023.2229070)
Supplement: Supplemental Material [file IENZ_A_2229070_SM9620.pdf]

Supporting Information

NMR Data

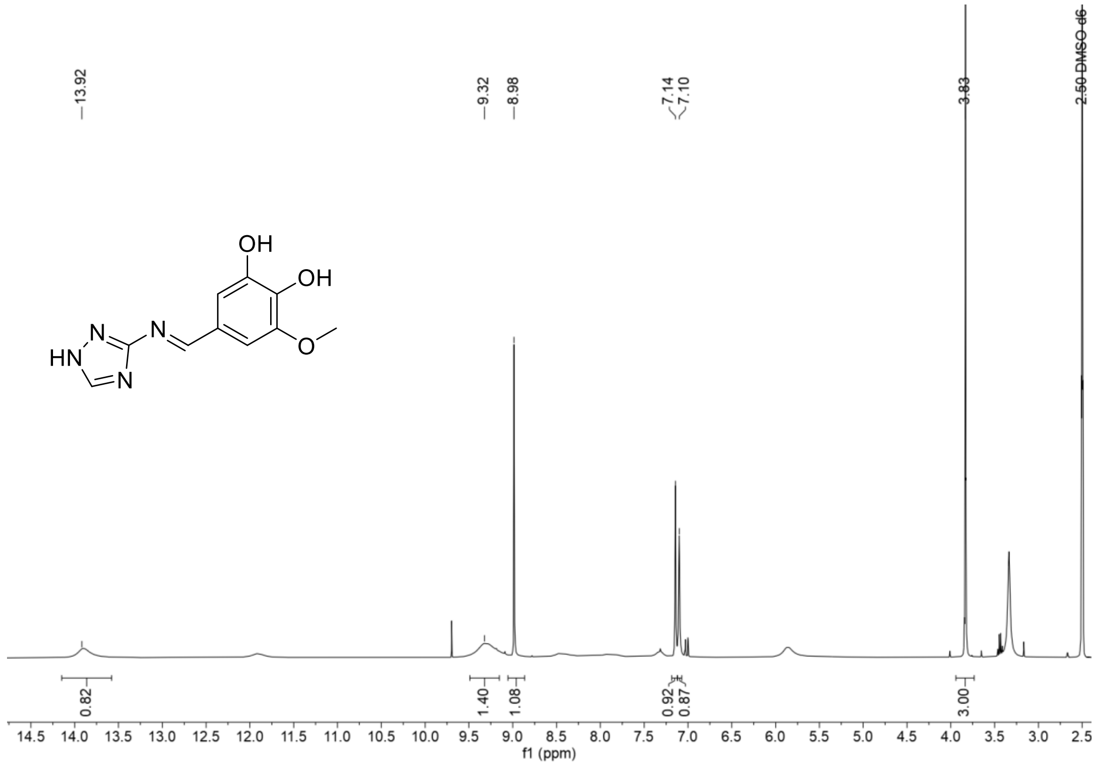

Figure S1. ^{1}H NMR Spectrum of Compound **1**

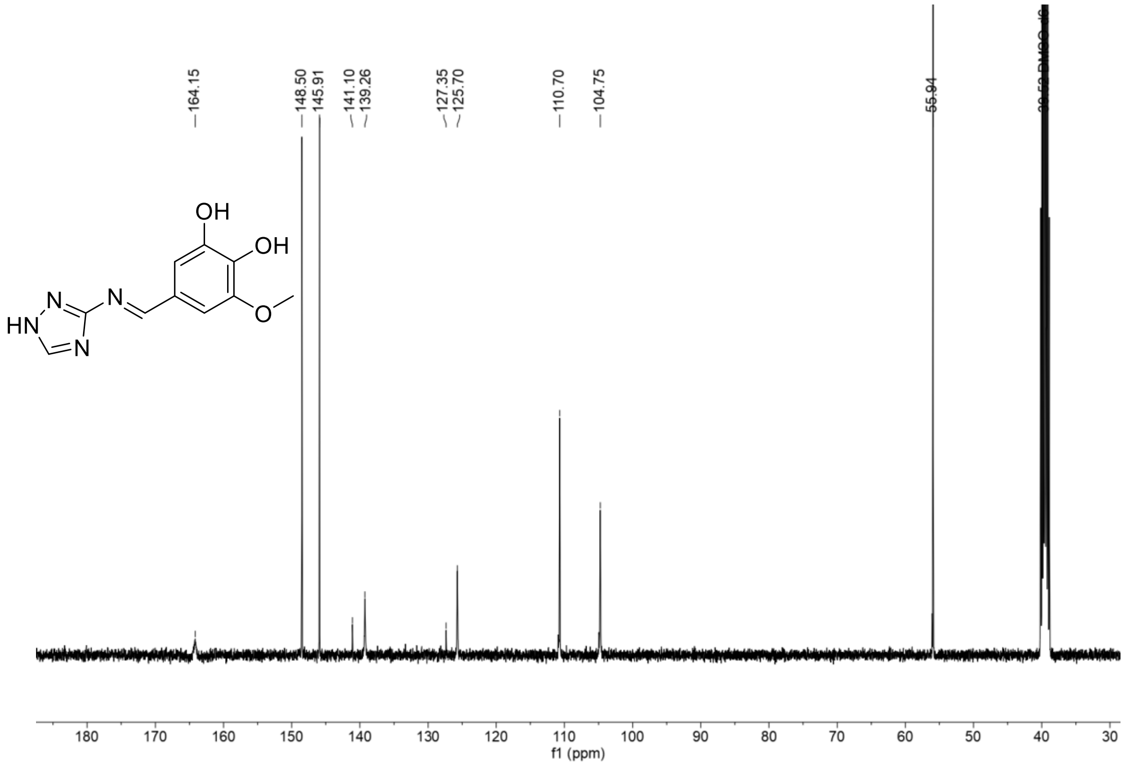

Figure S2. ^{13}C NMR Spectrum of Compound **1**

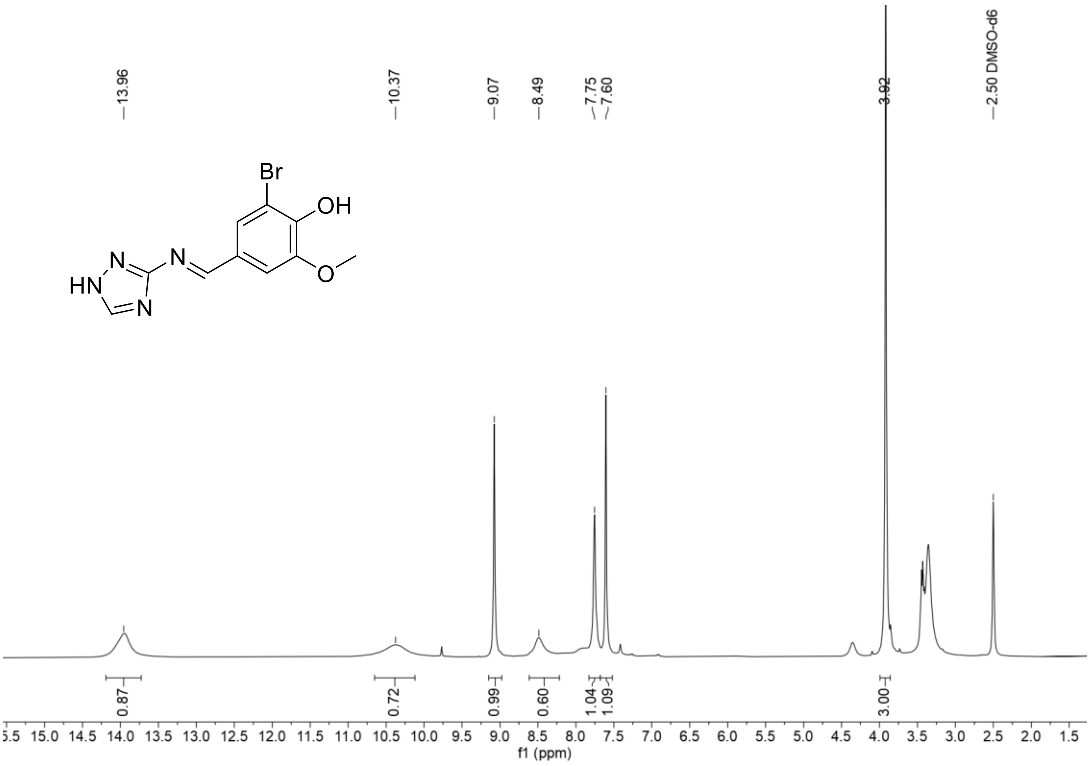

Figure S3. ^{1}H NMR Spectrum of Compound **2**

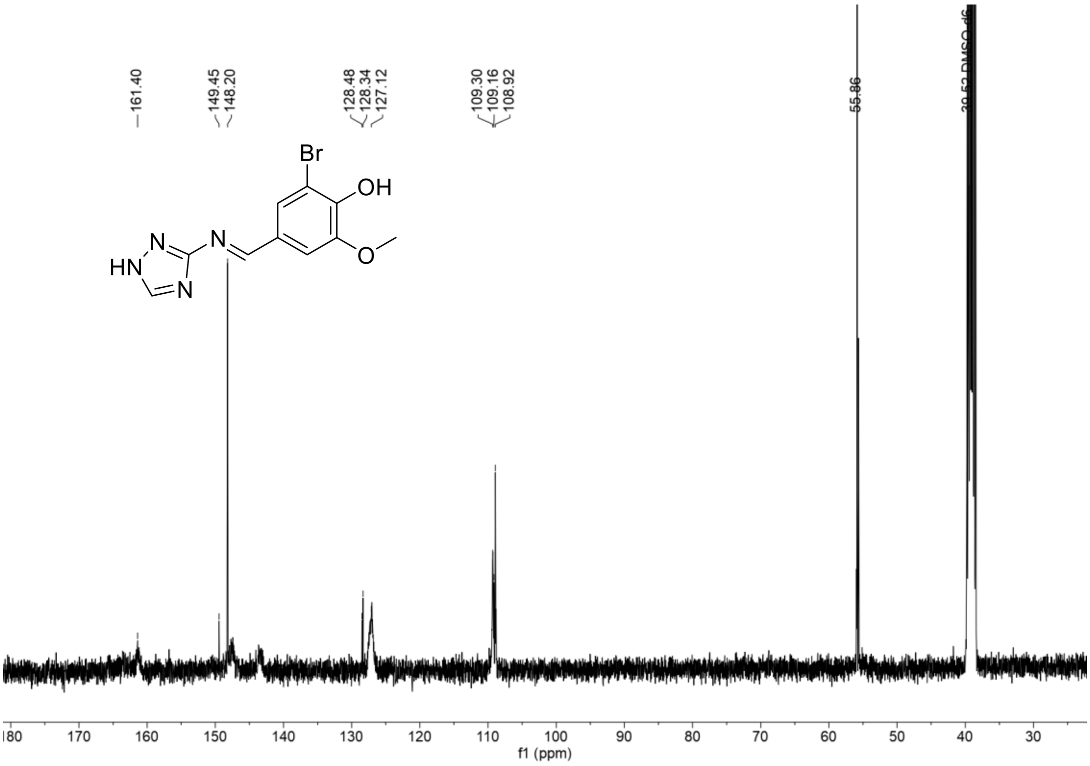

Figure S4. ^{13}C NMR Spectrum of Compound **2**

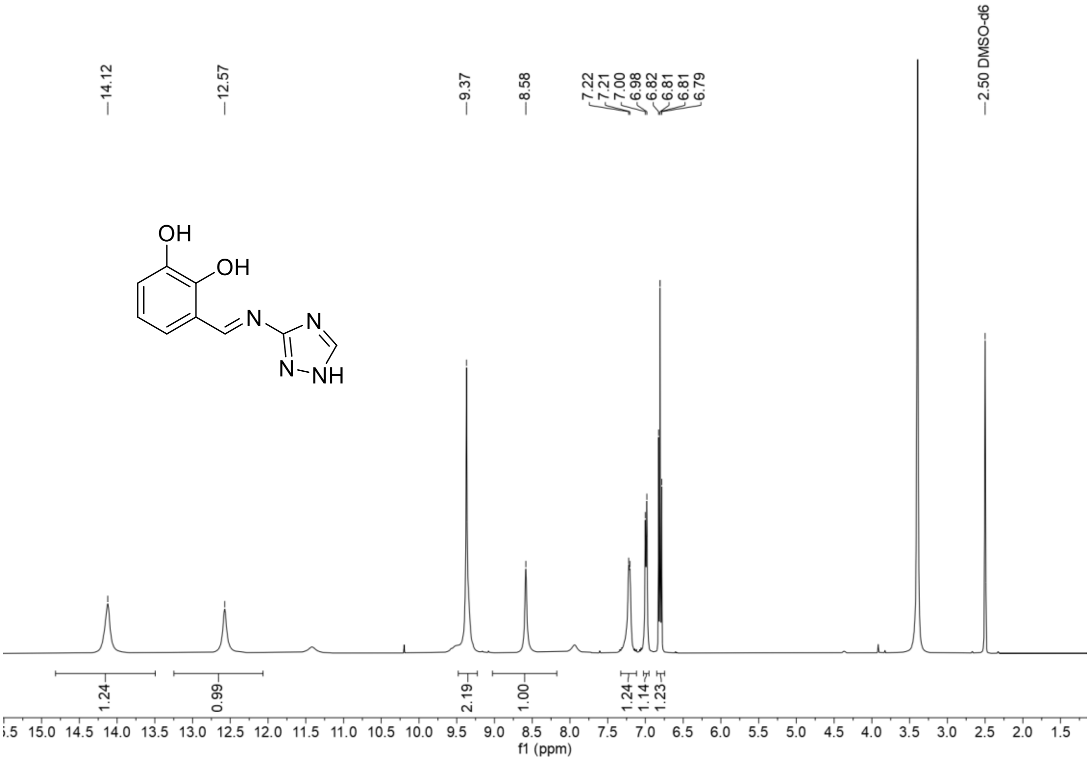

Figure S5. ^{1}H NMR Spectrum of Compound **3**

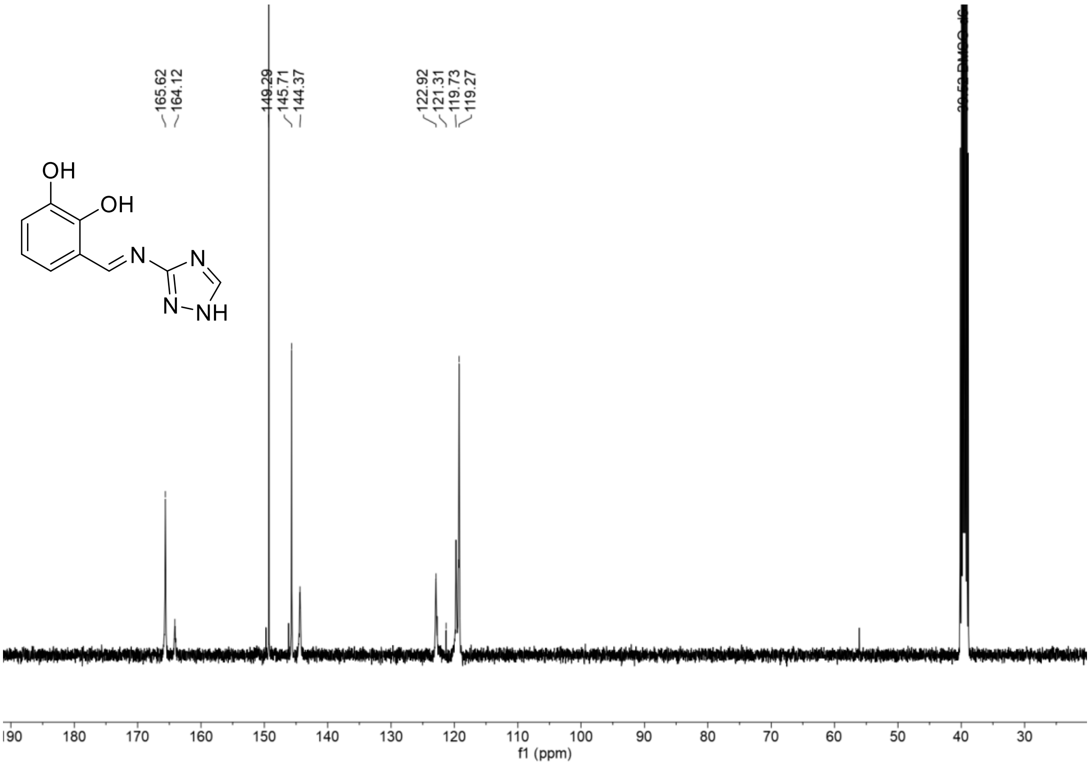

Figure S6. ^{13}C NMR Spectrum of Compound **3**

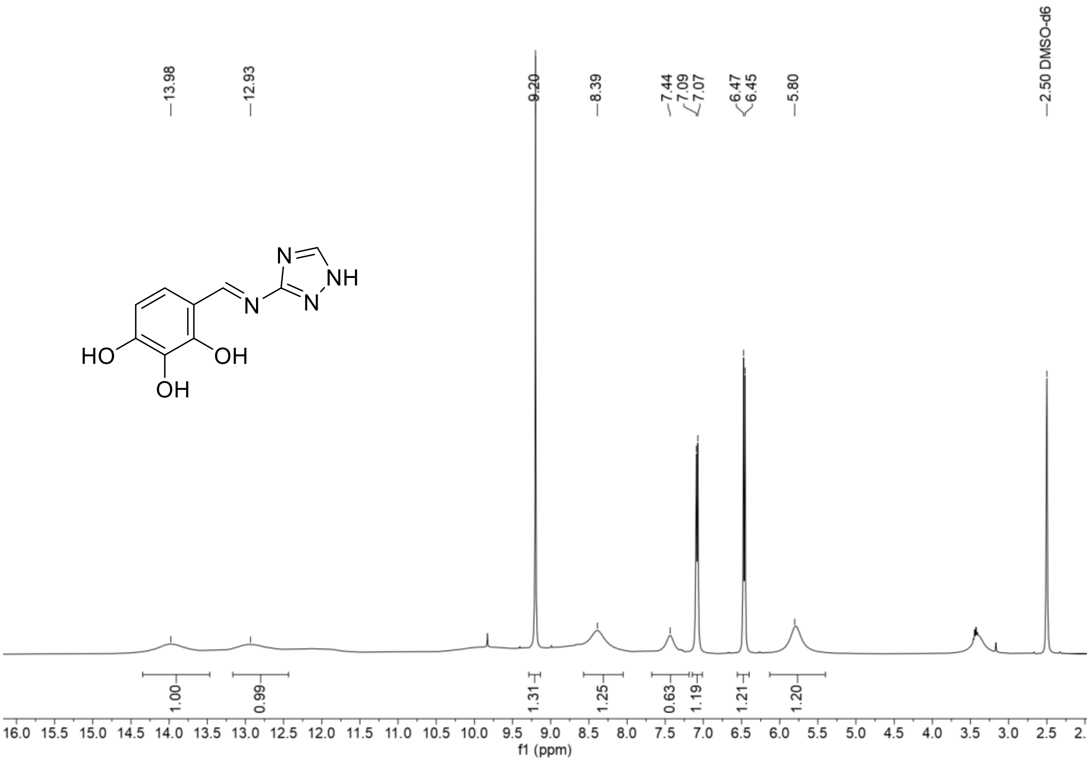

Figure S7. ^{1}H NMR Spectrum of Compound **4**

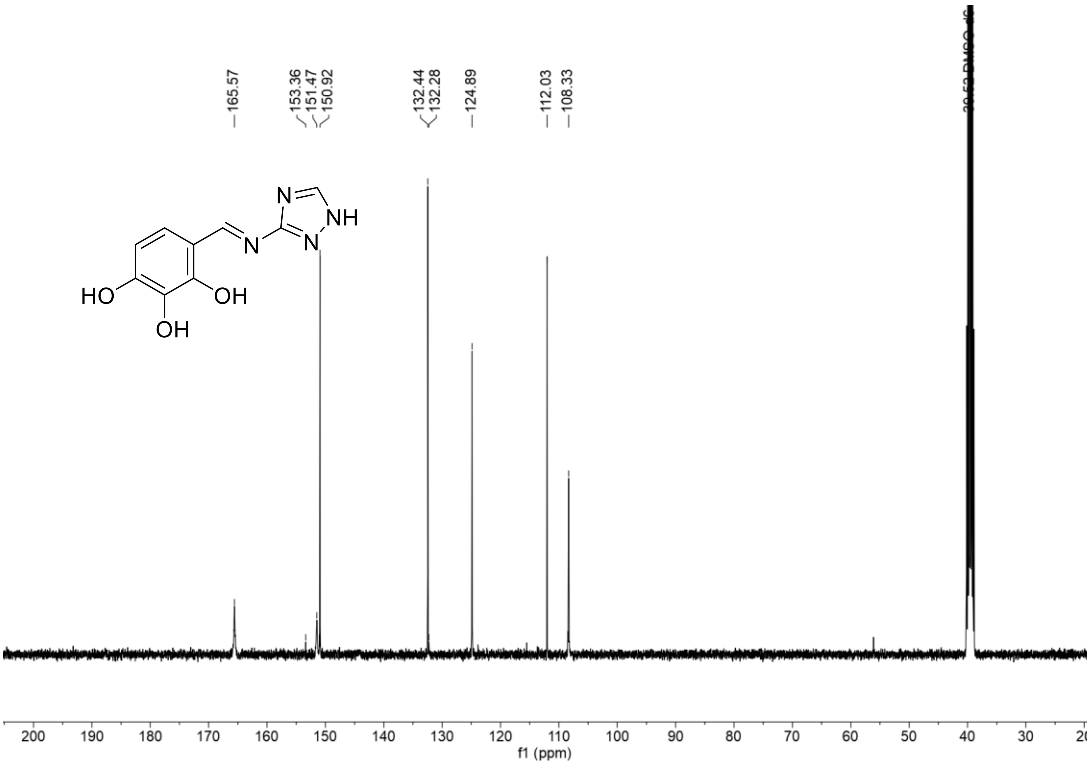

Figure S8. ^{13}C NMR Spectrum of Compound **4**

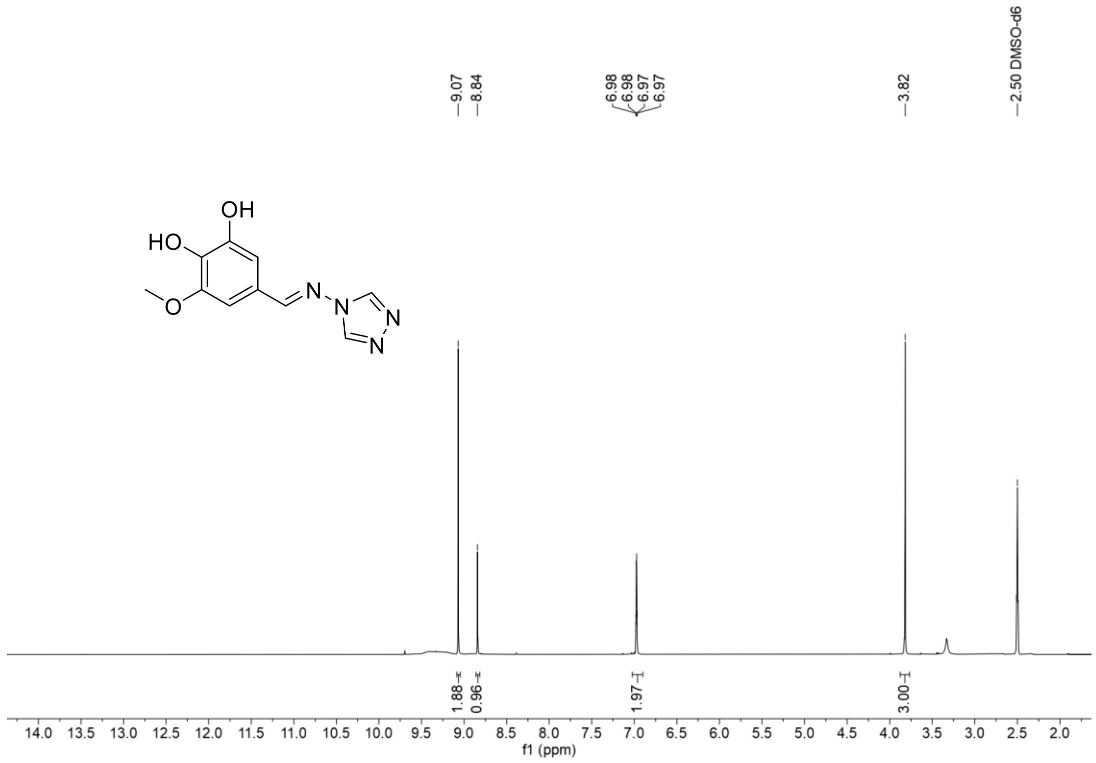

Figure S9. ^{1}H NMR Spectrum of Compound **5**

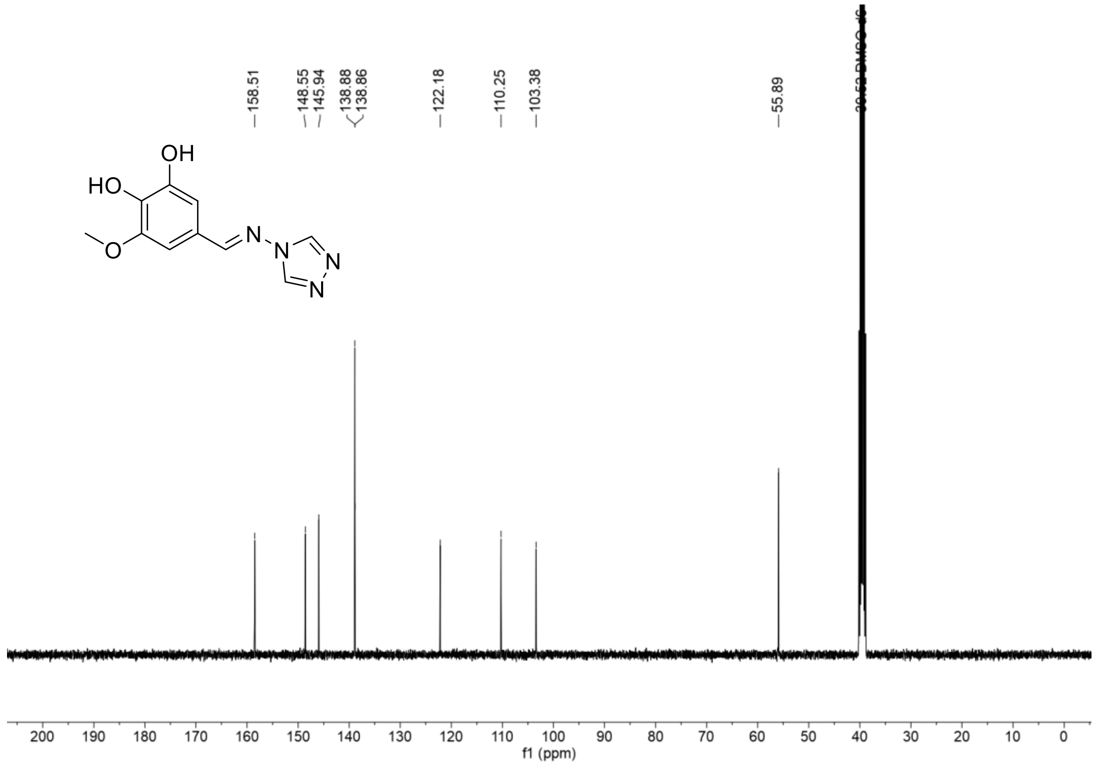

Figure S10. ^{13}C NMR Spectrum of Compound **5**

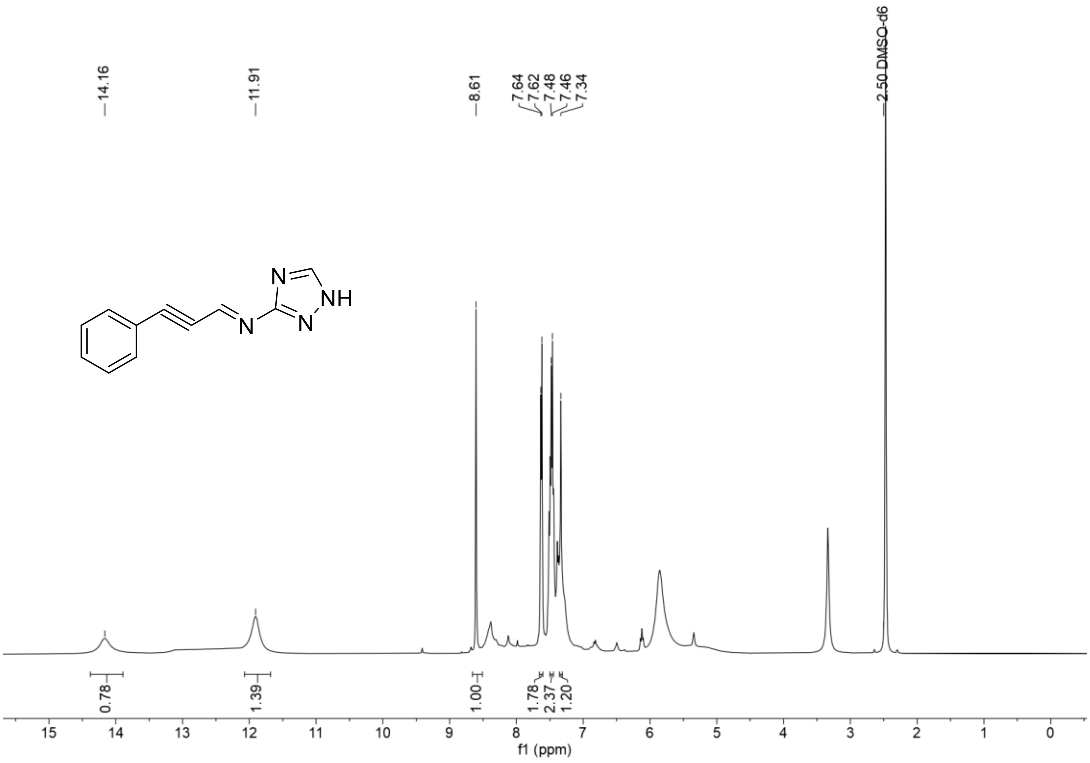

Figure S11. ^{1}H NMR Spectrum of Compound **6**

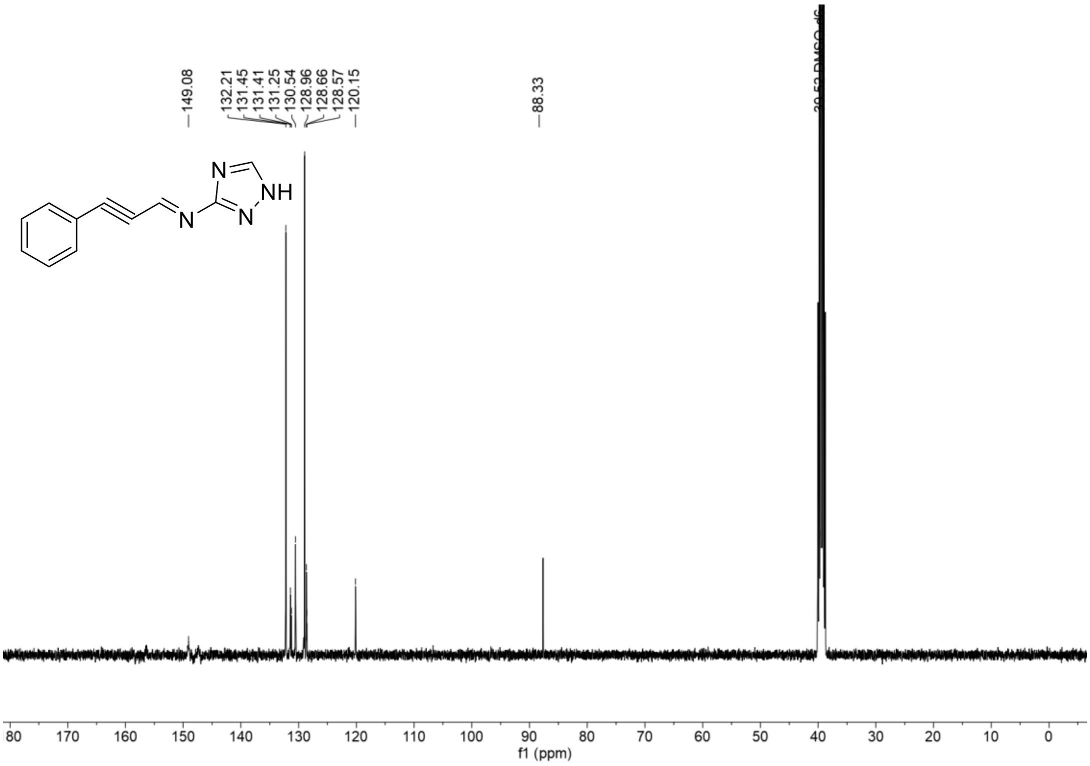

Figure S12. ^{13}C NMR Spectrum of Compound **6**

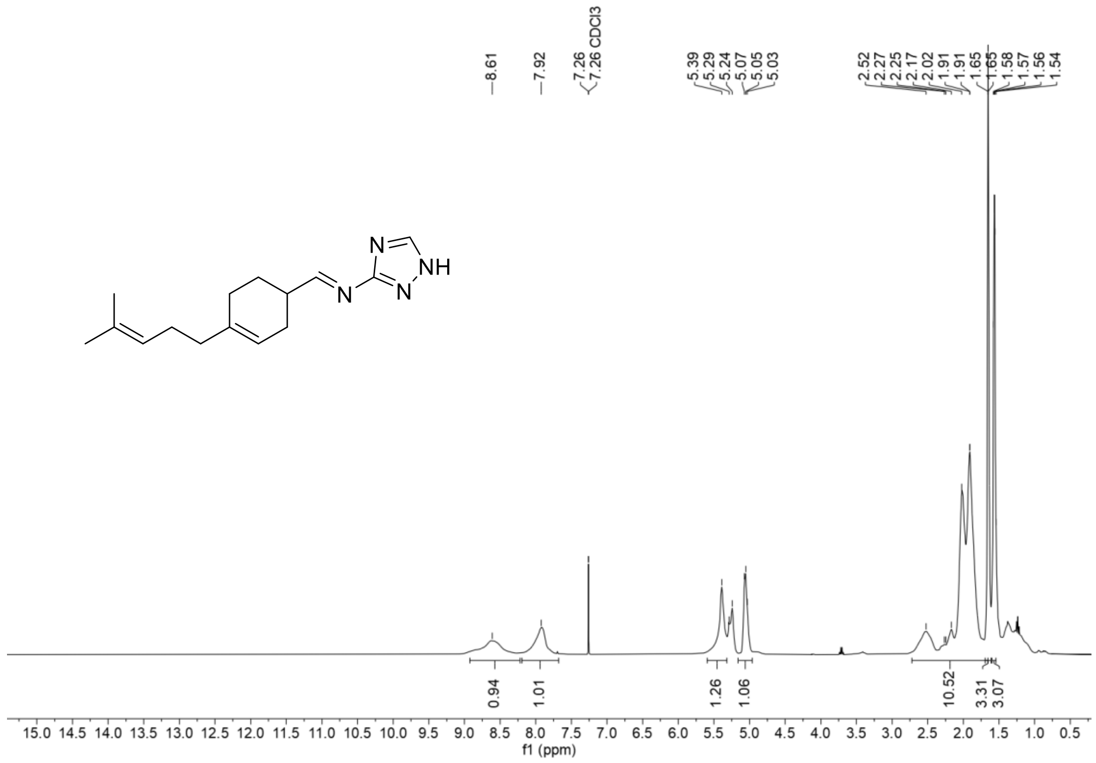

Figure S13. ^{1}H NMR Spectrum of Compound **7**

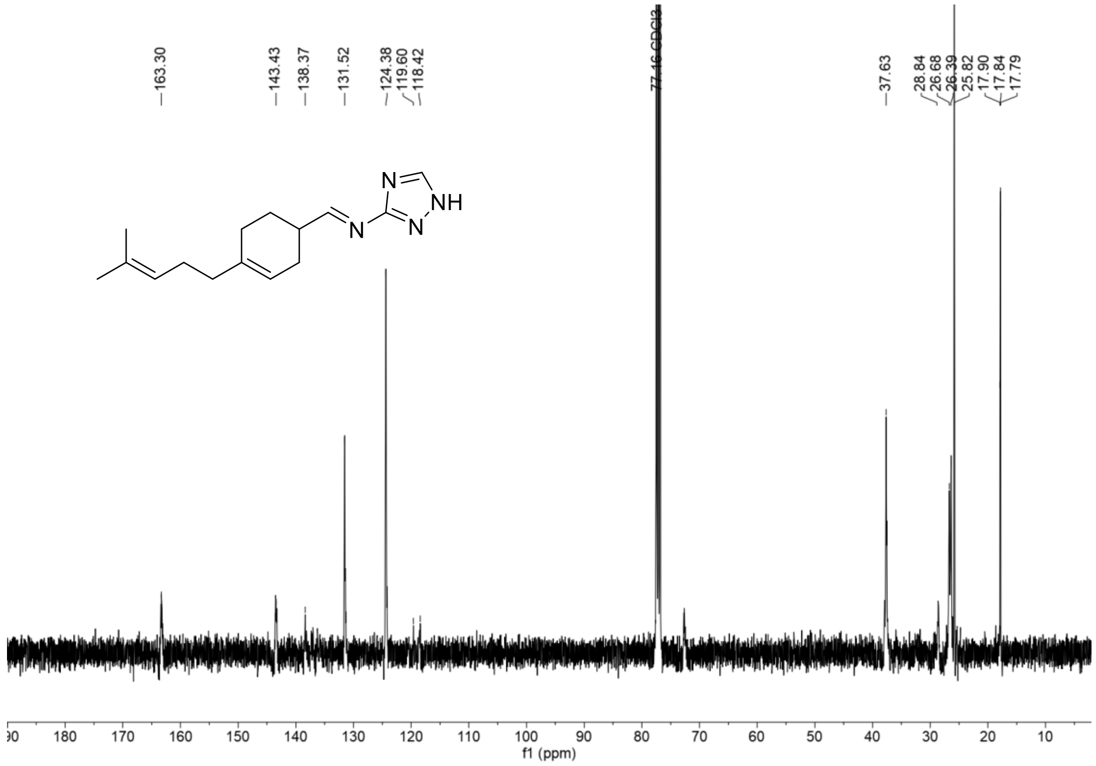

Figure S14. ^{13}C NMR Spectrum of Compound **7**

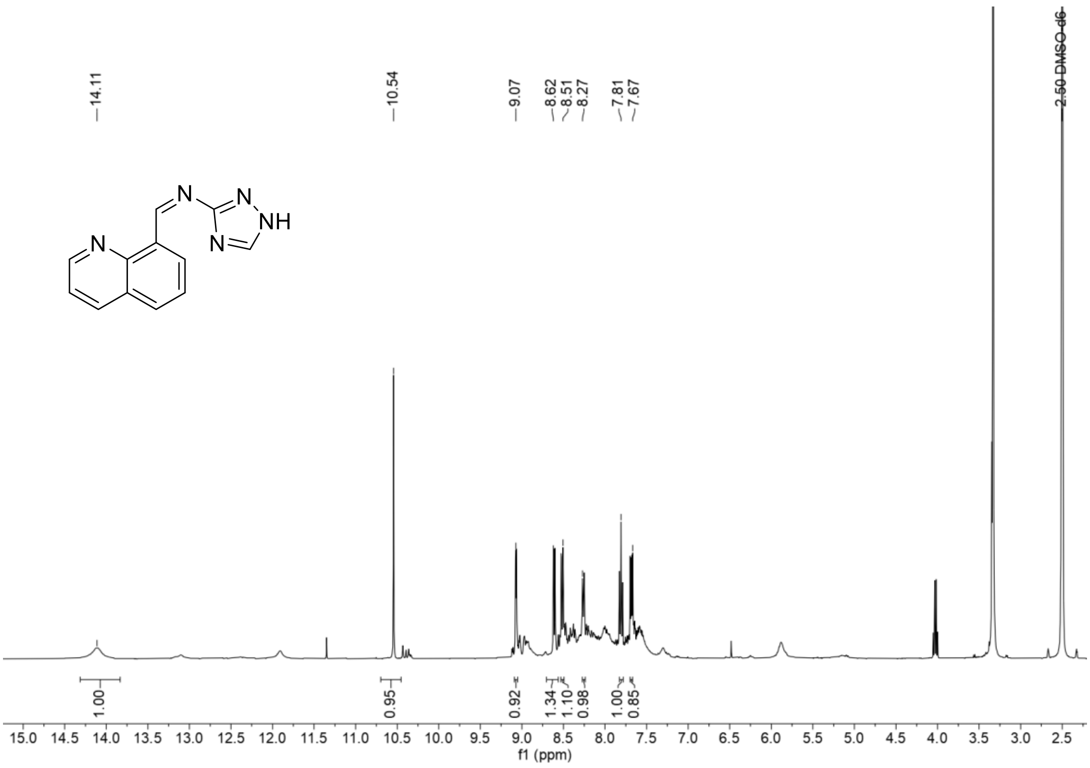

Figure S15. ^{1}H NMR Spectrum of Compound **8**

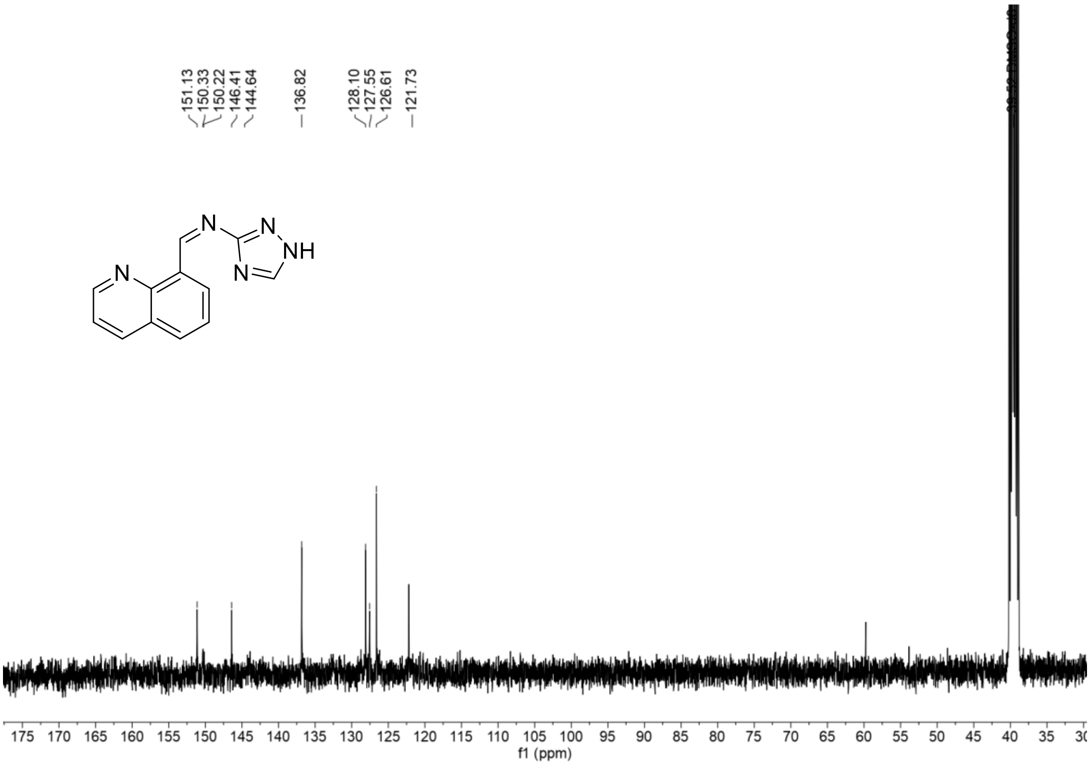

Figure S16. ^{13}C NMR Spectrum of Compound **8**

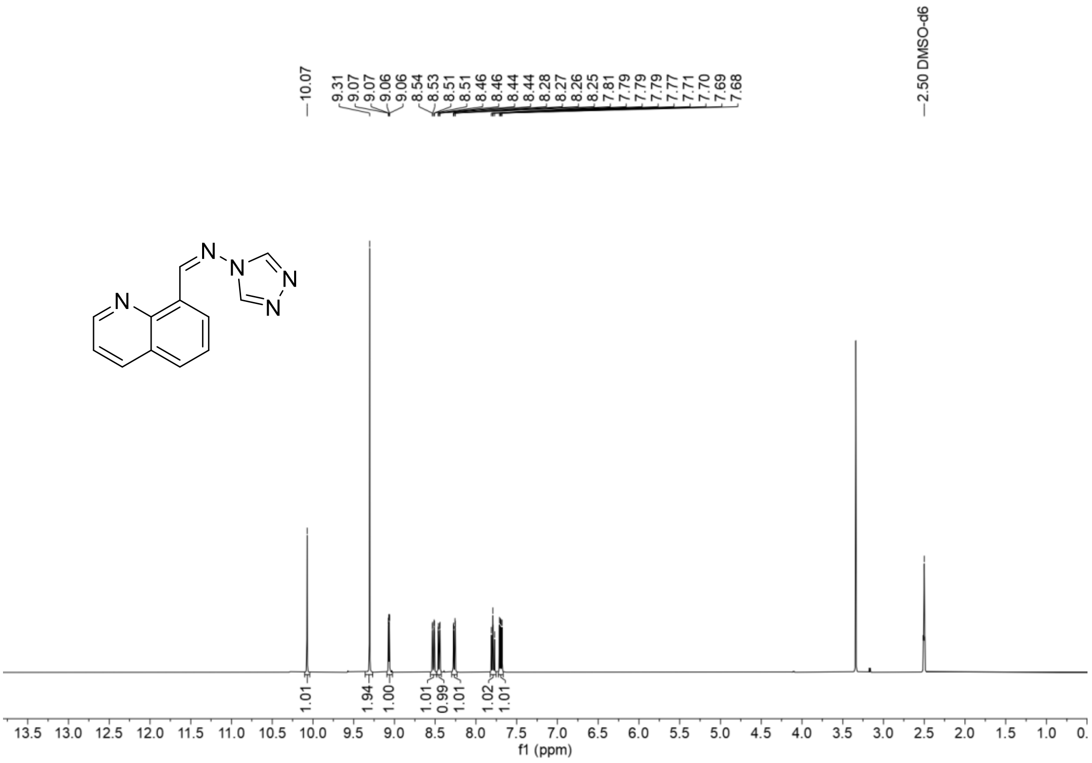

Figure S17. ^{1}H NMR Spectrum of Compound **9**

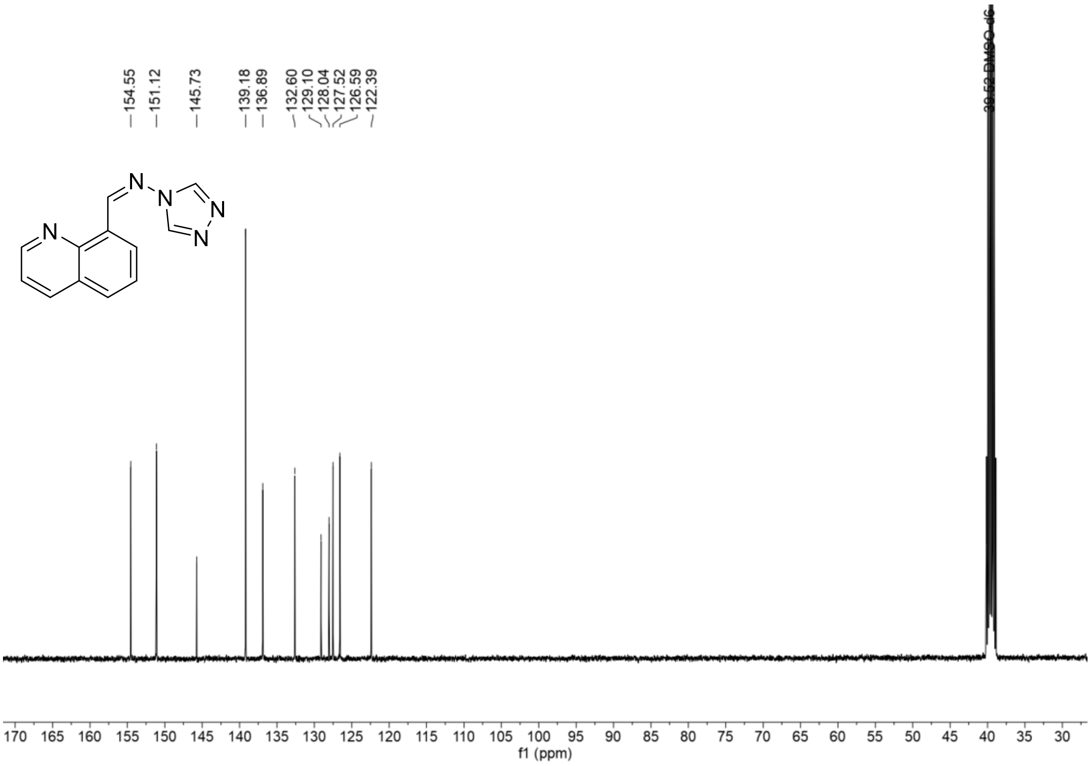

Figure S18. ^{13}C NMR Spectrum of Compound **9**

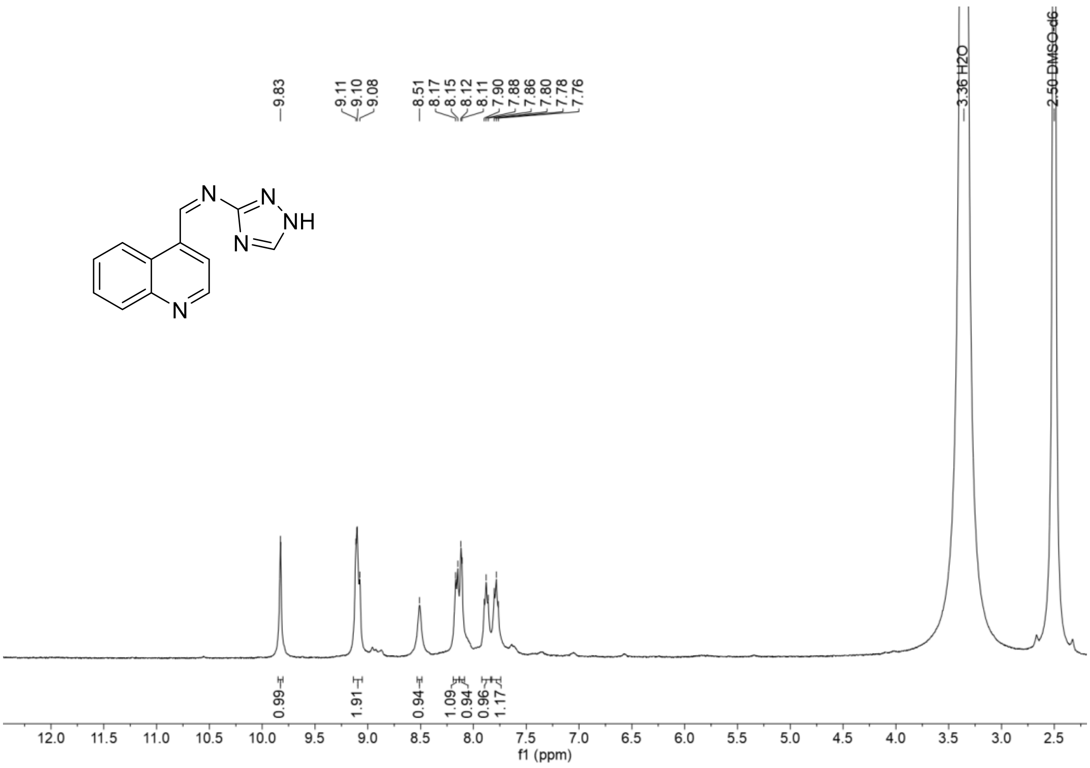

Figure S19. ^{1}H NMR Spectrum of Compound **10**

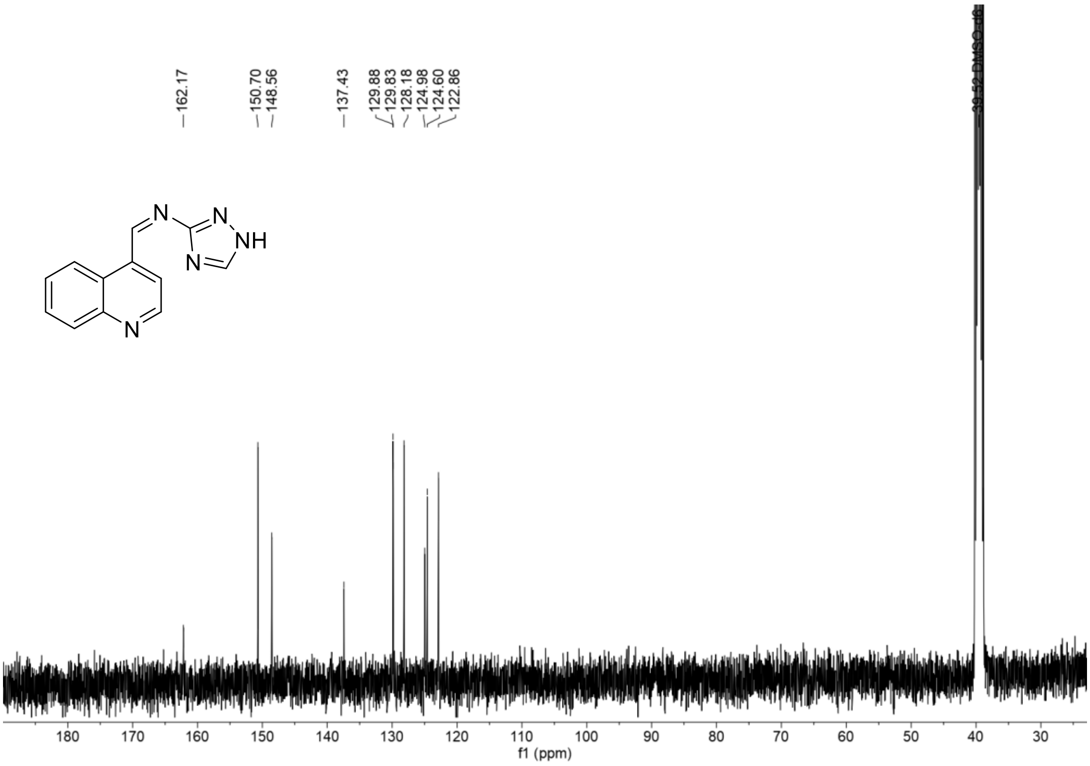

Figure S20. ^{13}C NMR Spectrum of Compound **10**

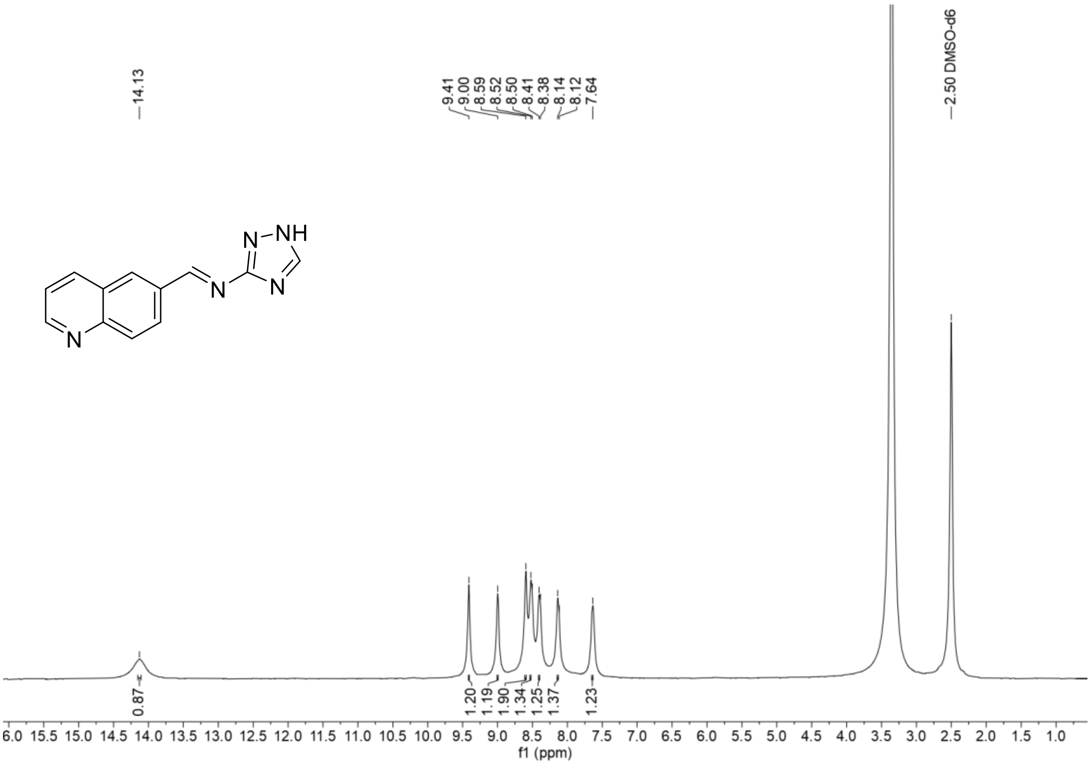

Figure S21. ^{1}H NMR Spectrum of Compound **11**

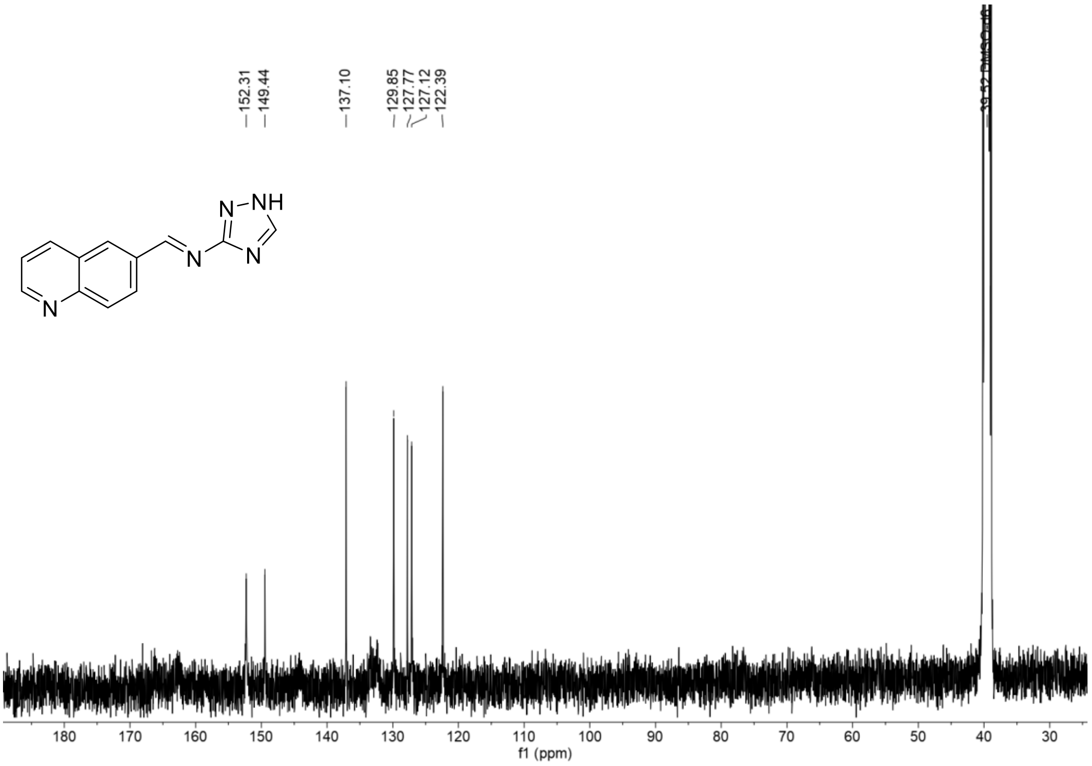

Figure S22. ^{13}C NMR Spectrum of Compound **11**

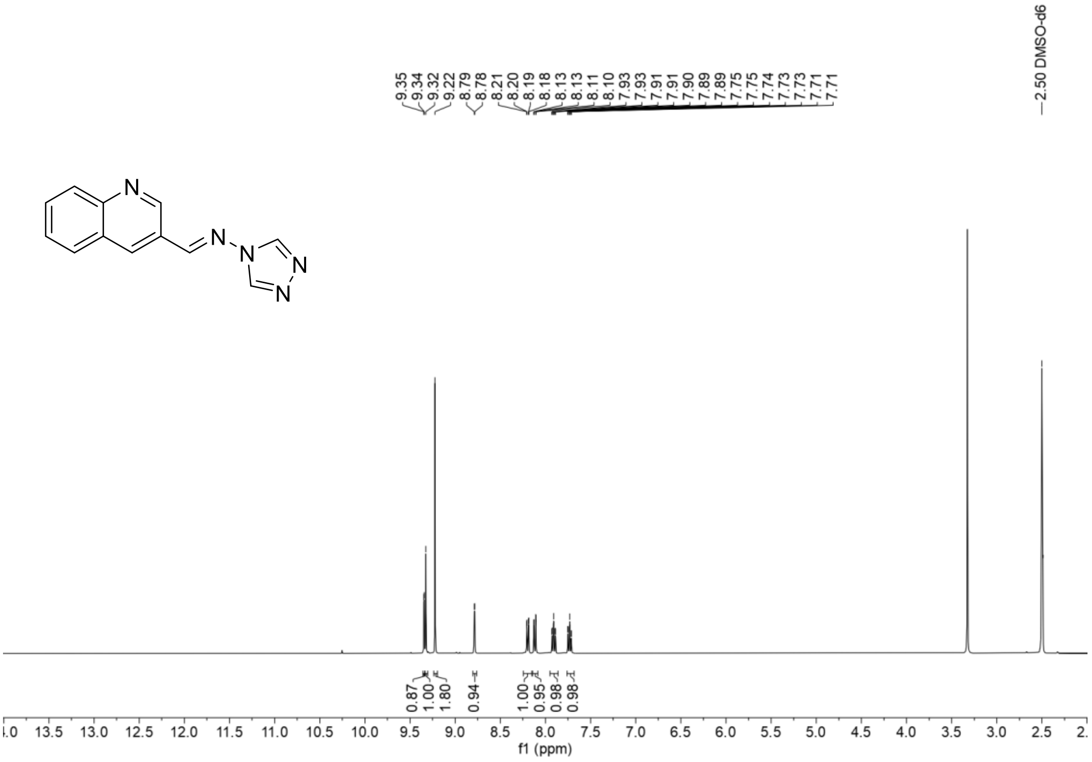

Figure S23. ^{1}H NMR Spectrum of Compound **12**

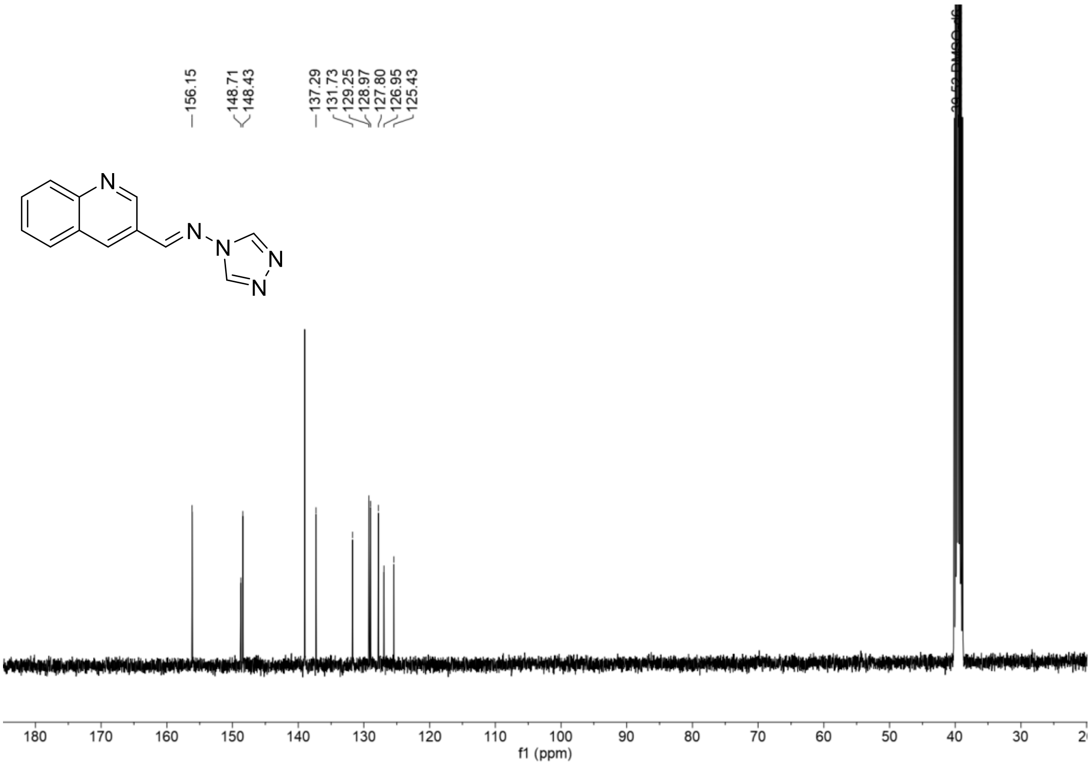

Figure S24. ^{13}C NMR Spectrum of Compound **12**

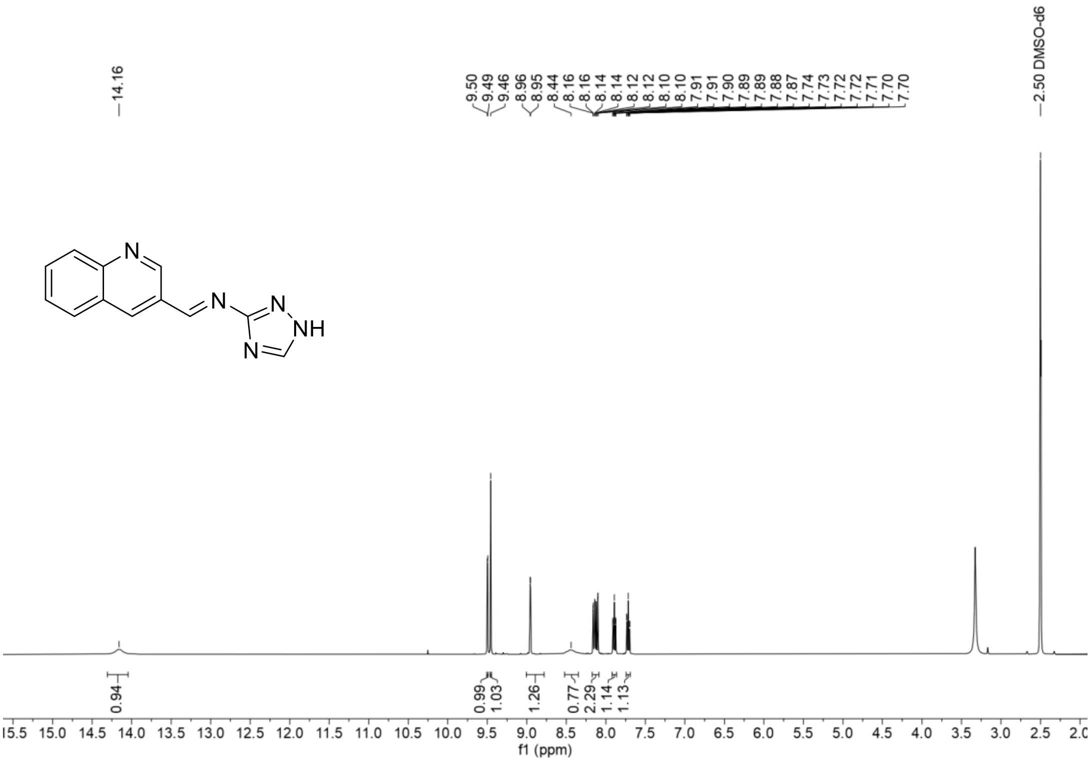

Figure S25. ^{1}H NMR Spectrum of Compound **13**

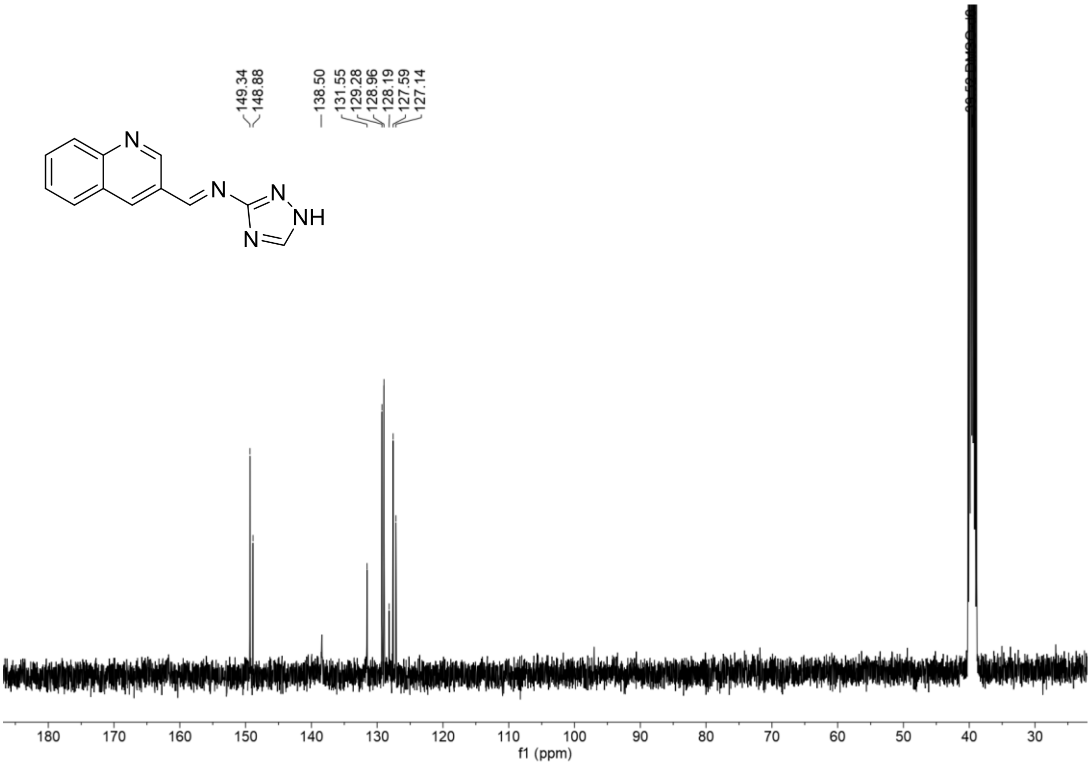

Figure S26. ^{13}C NMR Spectrum of Compound **13**

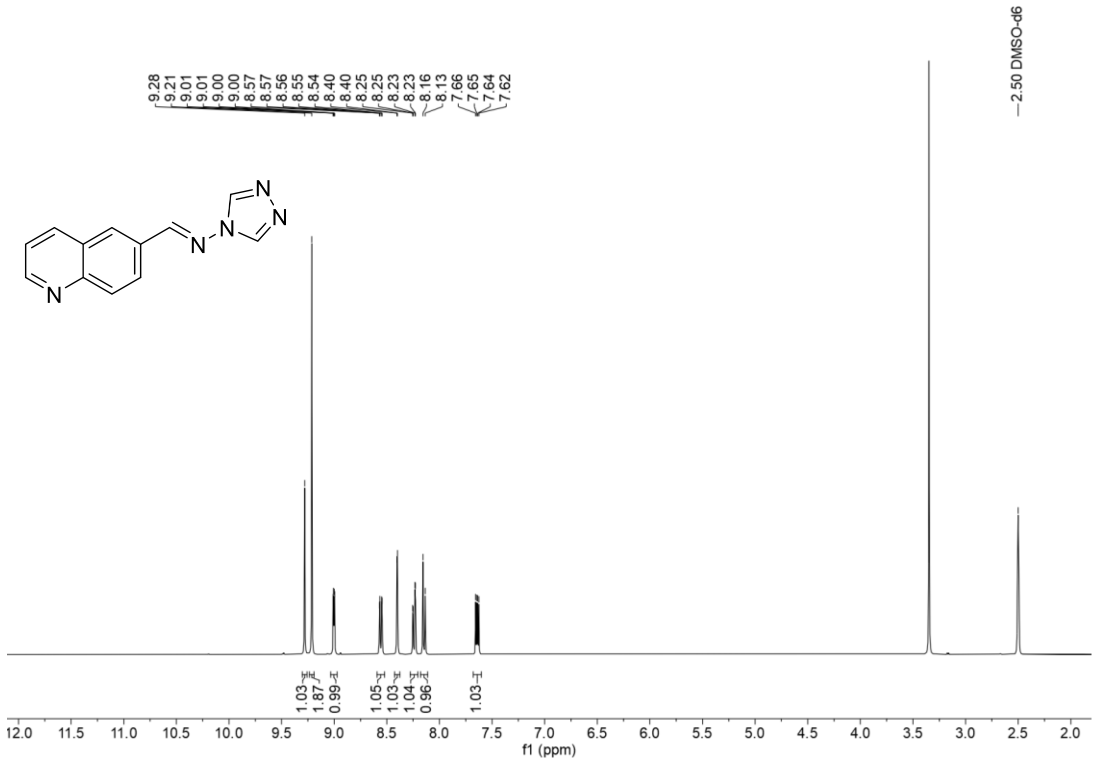

Figure S27. ^{1}H NMR Spectrum of Compound **14**

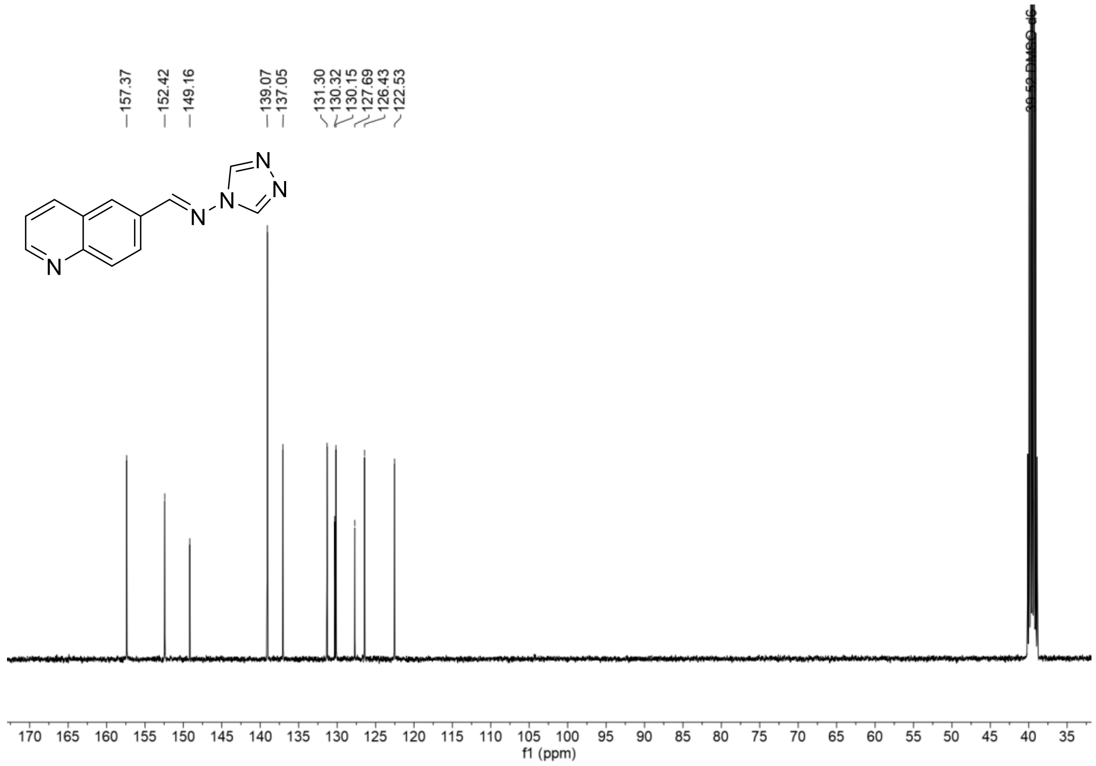

Figure S28. ^{13}C NMR Spectrum of Compound **14**

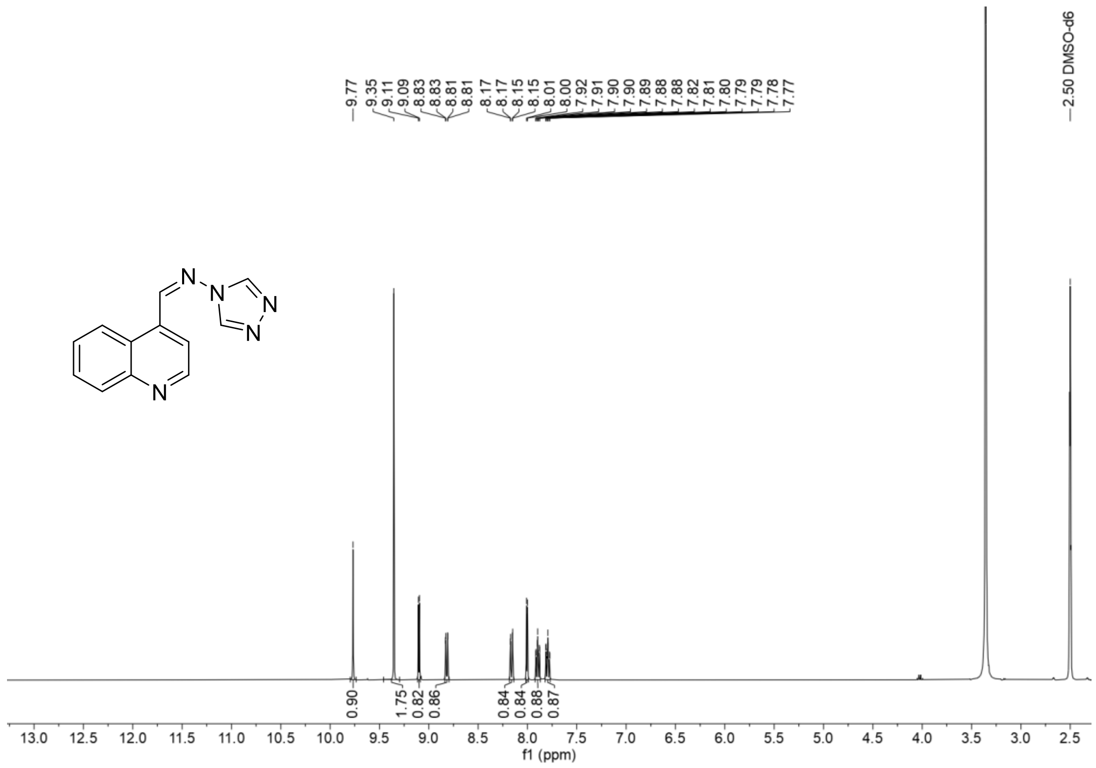

Figure S29. ^{1}H NMR Spectrum of Compound **15**

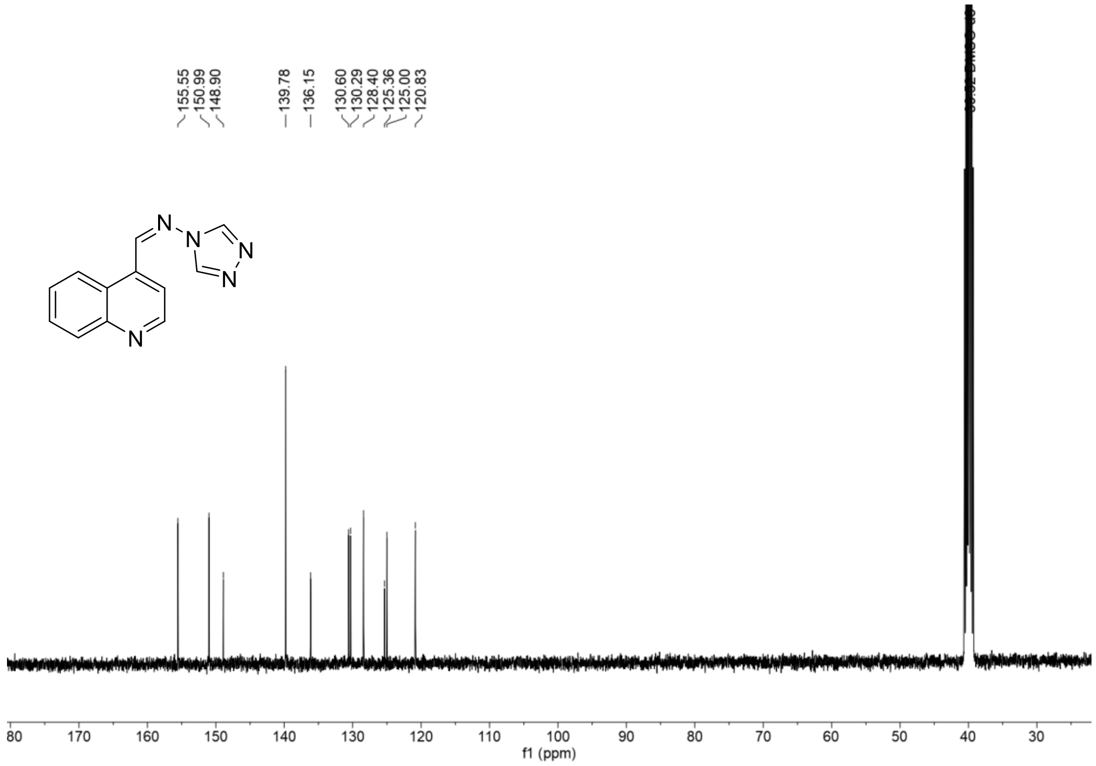

Figure S30. ^{13}C NMR Spectrum of Compound **15**
